# Automatic comprehensive aspects reports in clinical acute stroke MRIs

**DOI:** 10.1038/s41598-023-30242-6

**Published:** 2023-03-07

**Authors:** Chin-Fu Liu, Jintong Li, Ganghyun Kim, Michael I. Miller, Argye E. Hillis, Andreia V. Faria

**Affiliations:** 1 Center for Imaging Science, Johns Hopkins University, Baltimore, MD USA; 2 Department of Biomedical Engineering, Johns Hopkins University, Baltimore, MD USA; 3 Department of Physics, Johns Hopkins University, Baltimore, MD USA; 4 Department of Neuroscience, Johns Hopkins University, Baltimore, MD USA; 5 Department of Neurology, School of Medicine, Johns Hopkins University, Baltimore, MD USA; 6 Department of Physical Medicine and Rehabilitation, and Department of Cognitive Science, Johns Hopkins University, Baltimore, MD USA; 7 Department of Radiology, School of Medicine, Johns Hopkins University, Baltimore, MD USA

**Keywords:** Health care, Medical research, Neurology

## Abstract

The Alberta Stroke Program Early CT Score (ASPECTS) is a simple visual system to assess the extent and location of ischemic stroke core. The capability of ASPECTS for selecting patients’ treatment, however, is affected by the variability in human evaluation. In this study, we developed a fully automatic system to calculate ASPECTS comparable with consensus expert readings. Our system was trained in 400 clinical diffusion weighted images of patients with acute infarcts and evaluated with an external testing set of 100 cases. The models are interpretable, and the results are comprehensive, evidencing the features that lead to the classification. This system adds to our automated pipeline for acute stroke detection, segmentation, and quantification in MRIs (ADS), which outputs digital infarct masks and the proportion of diverse brain regions injured, in addition to the predicted ASPECTS, the prediction probability and the explanatory features. ADS is public, free, accessible to non-experts, has very few computational requirements, and run in real time in local CPUs with a single command line, fulfilling the conditions to perform large-scale, reproducible clinical and translational research.

## Introduction

The Alberta Stroke Program Early CT Score (ASPECTS) is a visual evaluation system to assess the extent and location of ischemic core in patients with acute strokes. Due to its relative simplicity of assessment, ASPECTS gained popularity and was also adapted to diffusion weighted MRIs (DWI)^1^. However, the capability of ASPECTS for selecting patients’ treatment is debatable^2,3^. A plausible reason might be the relative arbitrariness in human visual evaluation, especially when done by readers with less experience^4–6^. Recently, several automated methods have attempted to produce electronic scores (e-ASPECTS), and some have achieved comparable results to expert reading^7–10^.

All these systems, however, encountered challenges related to the ambiguous relation between visual and automated scores, due to the biological and technical variability. From the biological point of view, the variability in human evaluation, even for trained readers, increases in lesions affecting a small proportion of a given region, or peripherally located. From the technical point of view, the linear mapping of low-resolution clinical images to a common space, particularly in populations with substantial amounts of anatomical variability (e.g., elderly people) might lead to imprecision in the boundaries of the region of interest (ROI) and other specific areas (e.g., periventricular). Therefore, the visual and automated metrics need to be linked by models of order higher than a univariate correlation between the percentage of the lesion-affected ROI and the score.

In addition, for practical relevance, the automated systems for ASPECTS calculation have to be evaluated in large and independent clinical samples. They have to be readily accessible to users and report in real time. Finally, as any other machine learning (ML) development, the popularity of automated systems highly depends on their degree of interpretability^11,12^, i.e., in a comprehensive exposition of how the features used by ML models contribute to their predictions.

In this study, we developed an automatic ML system to calculate ASPECTS comparable to consensus expert readings on acute DWI. The results are comprehensive, showing the features that lead to the classification. This method adds on to our automated pipeline for acute stroke detection, segmentation, and quantification in MRIs (ADS^13^). Different from other systems, ADS is free, accessible to non- experts, running in local CPUs with a single command line and very few computational requirements, and outputting the results in real time. In addition to volumetric measures, ADS outputs the digital lesion segmentation, the brain images and lesion masks mapped to standard space, allowing the examination of the overlap of the lesion with specific brain structures, therefore granting crucial and objective quantitative information with broad access to the research community. The addition of an efficient ASPECTS calculation indicates that our fully automated system is able to extract personalized information of potential clinical relevance from stroke MRIs as efficiently as human experts do. This potentially sets the ground for the development of further computational-aids, such as electronic radiological reports^14^.

## Results

**Figure 1:**
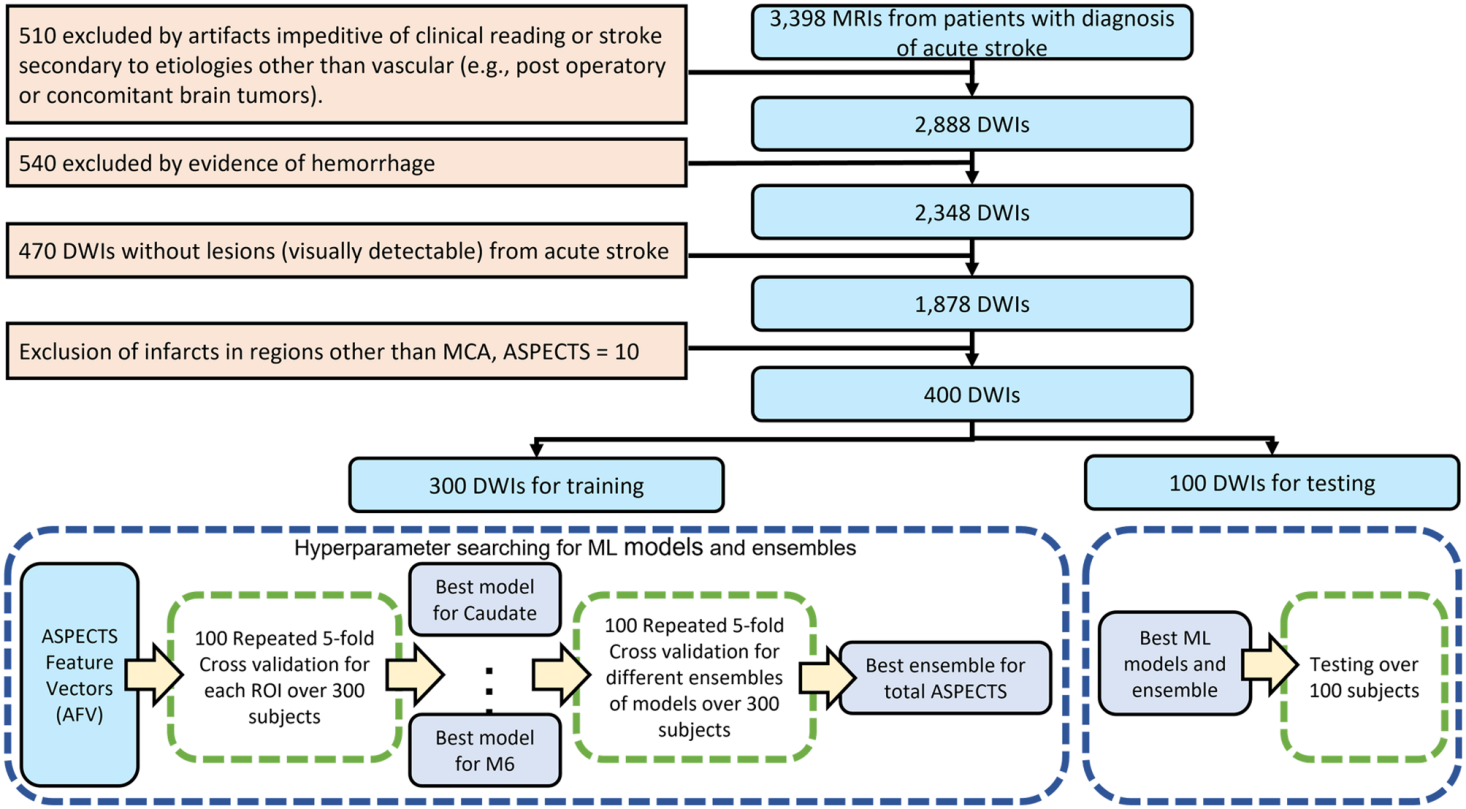
Flowchart of data inclusion (top) and training and testing sets for ML models (bottom).

**Figure 2:**
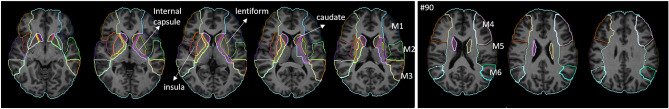
The ASPECTS ATLAS. The regions of interest (ROIs) are overlaid in the template T1-WI.

**Figure 3:**
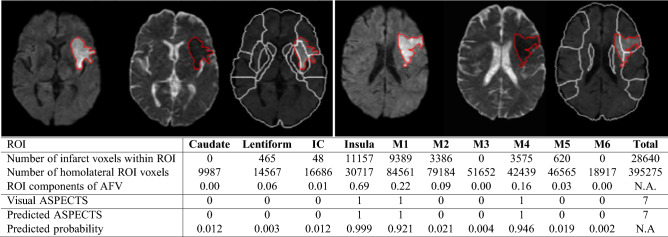
Example of ASPECTS feature vector (AFV) calculation. Each AFV component represents the percentage of the ASPECTS ROI affected by the infarct (number of voxels in which the infarct mask = 1/total number of voxels within the respective bilateral ROIs). The “visual ASPECTS” row shows the ASPECTS according consensus human evaluation, which is here considered the gold standard. The bottom two rows show the ASPECTS predicted by ML and the predicted probability, which are outputs of our automated tool, ADS.

The dataset included in this study (flowchart for data inclusion in Fig. 1) was random split into training (n = 300) and testing set (n = 100); the data profile is shown in Table 1. An ASPECTS atlas (Fig. 2 was created as a consistent framework for the visual analysis, as detailed in Methods. We used the proportion of ASPECTS ROIs affected by the infarct as the ASPECTS feature vectors (the "AFVs", Fig. 3) to train the ML models to predict ASPECTS. The details about the dataset and inclusion criteria, the ASPECTS atlas, the calculation of AFVs, the models’ training, optimization, and testing are in 'Methods' section.

**Table 1:** Population, lesion and scanner profiles. The table shows means or counts [interquartile range]. There were no statistical significant differences in distributions between testing and training sets, as shown in the "*p* value" column.

Dataset	Training	Testing	*p* value
Number of subjects	300	100	
Age in years	63 [53, 74]	60.5 [50.75, 72.25]	0.277
Sex			
Female	149	46	0.603
Male	151	54	
Race/ethnicity			
African American	165	52	0.783
Caucasian	116	40	
Asian	6	3	
Not recorded	13	5	
Hours from symptomns to MRI			
< 2	26	7	0.817
2–6	29	11	
6–12	30	19	
12–24	87	22	
> 24	21	3	
Not recorded	107	38	
Lesioned hemisphere			
Left	151	52	0.793
Right	149	47	
Bilateral	0	1	
Lesion volume in log ml	1.42 [0.99, 1.85]	1.39 [0.80, 1.90]	0.675
Scan manufacturer			
Siemens	271	93	0.181
Philips	3	0	
GE	21	3	
Other	5	4	
MRI magnetic field			
1.5 T	193	66	0.856
3.0 T	107	34	
Voxel size in mm^3^			
Volume	5.7 [3.58, 7.60]	5.7 [2.33, 7.20]	0.164
Height/width	1.20 [0.90, 1.30]	1.20 [0.60, 1.20]	
Thickness	5.00 [4.00, 5.00]	5.00 [4.00, 5.00]

### Correlations of visual ASPECTS and AFVs

Table 2a illustrates how ASPECTS attributed (by humans) to different ROIs relate to each other. High correlation between a pair ASPECTS ROIs’ mean they tend to co-exist in the population. As expected, given the spatial coalescence of infarcts, ASPECTS in neighboring ROIs were highly correlated (e.g., caudate–lentiform, insula–M5, M1–M4, M2–M5, and M3–M6). The lowest "neighboring" correlations were found in internal capsule, IC. The IC also showed the lowest inter-evaluator scoring agreement (Balanced Accuracy, BACC = 0.768), likely due to the challenges of visual analysis in this region, leading to increased variability in human classification.

**Table 2:** Correlation matrices of the visual ASPECTS and the ASPECTS feature vectors, AFVs. Note the high correlation in spatially neighbor regions found between visual ASPECTS (a) and between AFVs (b). Note that the highest correlation between visual ASPECTS and AFVs (c) is mostly found in the corresponding highest AFV component for each region, followed by the spatially neighboring regions.

	Caudate	Lentiform	IC	insula	M1	M2	M3	M4	M5	M6	Total ASPECTS
(a) ASPECTS v.s. ASPECTS											
Caudate	1.000	0.509	0.343	0.178	0.158	0.152	0.062	0.194	0.112	− 0.005	− 0.520
Lentiform	0.509	1.000	0.148	− 0.126	0.009	− 0.023	− 0.054	0.024	− 0.128	− 0.126	− 0.241
IC	0.343	0.148	1.000	− 0.081	− 0.011	0.029	− 0.004	0.045	− 0.025	0.016	− 0.246
Insula	0.178	− 0.126	− 0.081	1.000	0.406	0.394	0.205	0.380	0.523	0.162	− 0.625
M1	0.158	0.009	− 0.011	0.406	1.000	0.084	0.053	0.750	0.166	0.053	− 0.530
M2	0.152	− 0.023	0.029	0.394	0.084	1.000	0.389	0.166	0.603	0.344	− 0.641
M3	0.062	− 0.054	− 0.004	0.205	0.053	0.389	1.000	0.096	0.254	0.735	− 0.552
M4	0.194	0.024	0.045	0.380	0.750	0.166	0.096	1.000	0.130	0.049	− 0.561
M5	0.112	− 0.128	− 0.025	0.523	0.166	0.603	0.254	0.130	1.000	0.239	− 0.595
M6	− 0.005	− 0.126	0.016	0.162	0.053	0.344	0.735	0.049	0.239	1.000	− 0.496
Total ASPECTS	− 0.520	− 0.241	− 0.246	− 0.625	− 0.530	− 0.641	− 0.552	− 0.561	− 0.595	− 0.496	1.000

	AFV_Caudate	AFV_lentiform	AFV_IC	AFV_insula	AFV_M1	AFV_M2	AFV_M3	AFV_M4	AFV_M5	AFV_M6	Lesion volume
(b) AFV v.s. AFV											
AFV_Caudate	1.000	0.884	0.906	0.531	0.468	0.419	0.245	0.427	0.385	0.166	0.400
AFV_lentiform	0.884	1.000	0.933	0.662	0.502	0.529	0.351	0.460	0.467	0.261	0.486
AFV_IC	0.906	0.933	1.000	0.568	0.429	0.470	0.321	0.409	0.425	0.244	0.396
AFV_insula	0.531	0.662	0.568	1.000	0.744	0.777	0.488	0.638	0.702	0.404	0.769
AFV_M1	0.468	0.502	0.429	0.744	1.000	0.608	0.352	0.888	0.611	0.305	0.620
AFV_M2	0.419	0.529	0.470	0.777	0.608	1.000	0.799	0.575	0.821	0.664	0.777
AFV_M3	0.245	0.351	0.321	0.488	0.352	0.799	1.000	0.371	0.637	0.856	0.667
AFV_M4	0.427	0.460	0.409	0.638	0.888	0.575	0.371	1.000	0.615	0.339	0.620
AFV_M5	0.385	0.467	0.425	0.702	0.611	0.821	0.637	0.615	1.000	0.672	0.768
AFV_M6	0.166	0.261	0.244	0.404	0.305	0.664	0.856	0.339	0.672	1.000	0.631
Lesion volume	0.400	0.486	0.396	0.769	0.620	0.777	0.667	0.620	0.768	0.631	1.000

	AFV_Caudate	AFV_lentiform	AFV_IC	AFV_insula	AFV_M1	AFV_M2	AFV_M3	AFV_M4	AFV_M5	AFV_M6	Lesion volume
(c) ASPECTS v.s. AFV											
Caudate	0.830	0.746	0.764	0.441	0.303	0.313	0.168	0.267	0.264	0.086	0.315
Lentiform	0.597	0.674	0.675	0.207	0.147	0.130	0.060	0.136	0.062	− 0.031	− 0.033
IC	0.381	0.381	0.482	0.132	0.115	0.121	0.099	0.114	0.117	0.078	− 0.006
Insula	0.200	0.283	0.205	0.708	0.463	0.498	0.284	0.411	0.506	0.264	0.606
M1	0.245	0.285	0.198	0.536	0.750	0.320	0.127	0.691	0.357	0.113	0.466
M2	0.209	0.299	0.256	0.506	0.323	0.744	0.546	0.342	0.634	0.438	0.591
M3	0.092	0.190	0.142	0.315	0.174	0.565	0.828	0.198	0.418	0.689	0.536
M4	0.274	0.310	0.251	0.520	0.685	0.342	0.178	0.754	0.391	0.153	0.477
M5	0.195	0.260	0.198	0.517	0.323	0.589	0.365	0.304	0.721	0.360	0.578
M6	0.046	0.135	0.086	0.247	0.171	0.492	0.745	0.189	0.440	0.829	0.521
Total ASPECTS	− 0.597	− 0.700	− 0.633	− 0.829	− 0.688	− 0.830	− 0.683	− 0.678	− 0.790	− 0.599	− 0.819

Table 2b demonstrates the correlation between AFV components. It represents the quantitative version of the qualitative scores in Table 2a, to which it highly agrees. This indicates that the quantitative information encoded in the AFVs (the proportion of each ROI affected by the infarct) is likely reflecting the qualitative information that humans relay on for their visual analysis. As expected, each AFV component is highly correlated to the AFV components of its neighbor ROIs. We note that the correlations between AFVs tend to be higher than the correlations between visual ASPECTS, which is probably related to the continuous nature of the former.

**Figure 4:**
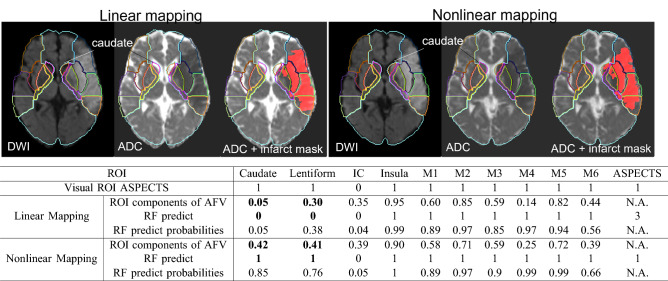
Illustrative example of prediction error related to brain mapping. Note that when the brain is mapped to the template by linear transformation, the agreement of internal structures with their atlas definition is sub-optimum. This is particularly important for brains with specific characteristics (e.g., hydrocephalus) and periventricular structures (e.g., caudate). The imprecision affects the calculus of the proportion of the region affected by the stroke and consequently, the prediction of ASPECTS. In this case, the linear brain mapping to the template (left panel) grades the caudate as 5% affected by the infarct and it consequently scores "0" (bold cells in the table). With non-linear mapping (right panel), the match between the brain and atlas increases; the caudate injury is now quantified as 42% and the predicted score is 1 (bold cells in the table), in agreement with the human evaluation. The same happened to Lentiform.

Table 2c combines the information above, showing the correlation between visual consensus ASPECTS and AFV components. It indicates more directly how humans inconspicuously use the quantitative information about the spatial distribution of the infarct lesion (reflected by the AFVs) to attribute ASPECTS. The rows of 2c indicate that the ASPECTS attributed to each ROI is mostly correlated to the AFV component that corresponds to the ROI in question, and secondly, to the AFV components corresponding to neighboring ROIs, as expected. As observed in the correlations between visual ASPECTS, as the infarct extends beyond the artificial limits of the areas semantically defined, the human evaluation is not purely based on how individual areas are affected, but also in the regional lesion pattern. Interestingly, the columns of Table 2c show that the greatest AFV component (which corresponds to the ROI mostly affected by the infarct) is not always firstly correlated with ASPECTS of the ROI in question. For example, the AFV-IC is more correlated to caudate visual ASPECTS than to IC visual ASPECTS. The same applies to AFV-lentiform. This again may reflect human challenges and variability to define infarction of regions as IC. It may also reflect imprecision in the linear brain mapping, affecting the alignment between the template and atlas to the lesion masks. This imprecision is particularly more noticeable around the caudate and IC due to common midline shifts caused by acute stroke edema and hydrocephalus, frequently observed in the stroke population (an illustrative example is shown in Fig. 4).

### Performance of ML models for ASPECTS prediction

The performance of the ML models to predict ASPECTS in the external testing set is summarized in Table 3. The performance in the cross-validation sample is shown in the Supplementary Table 3. The prediction of ASPECTS was comparable to inter-evaluators’ agreement in Balanced Accuracy (BACC) and F1 score (defined in Methods). The lowest agreement, while still satisfactory, occurred in the IC (aligned with the lowest inter-evaluators’ agreement). The second lowest agreement, compared to inter-evaluators, occurred in the caudate. Again, technical factors related to the linear mapping and specific characteristics of this population (e.g., hydrocephalus) may be responsible for the slightly low accuracy. The most efficient models were Random Forest (RF), Multi-layer Perceptron (MLP), and Support Vector Machine (SVM). These three best models were assembled to generate a final model that is included in our deployed pipeline to calculate ASPECTS in ADS^13^.

**Table 3:** Comparison of performance of ML models and inter-annotators in the external testing set (n = 100). For the total aspects "whit tolerance", predicted ASPECTS within ± 1 difference from the ground true ASPECTS were considered true positives.

ROI	Number of occurrences	Metric	LDA	QDA	KNN	SVM	RF	MLP	BT	Ensemble	Inter-evaluators
Caudate	36	BACC	0.853	0.839	0.822	0.832	0.850	0.818	0.878	0.832	0.901
		F1	0.825	0.789	0.776	0.794	0.812	0.774	0.845	0.794	0.882
		Precision	0.963	0.750	0.839	0.926	0.848	0.923	0.857	0.926	0.938
		Sensitivity	0.722	0.833	0.722	0.694	0.778	0.667	0.833	0.694	0.833
		Kappa	0.747	0.661	0.664	0.702	0.713	0.677	0.760	0.702	0.822
Lentiform	59	BACC	0.851	0.885	0.917	0.885	0.942	0.916	0.859	0.938	0.903
		F1	0.870	0.919	0.932	0.919	0.948	0.920	0.879	0.949	0.899
		Precision	0.893	0.877	0.932	0.877	0.965	0.963	0.895	0.949	0.980
		Sensitivity	0.847	0.966	0.932	0.966	0.932	0.881	0.864	0.949	0.831
		Kappa	0.693	0.789	0.835	0.789	0.877	0.817	0.713	0.876	0.780
IC	23	BACC	0.757	0.792	0.676	0.704	0.770	0.791	0.702	0.798	0.768
		F1	0.650	0.654	0.514	0.571	0.684	0.718	0.545	0.737	0.638
		Precision	0.765	0.586	0.750	0.833	0.867	0.875	0.571	0.933	0.625
		Sensitivity	0.565	0.739	0.391	0.435	0.565	0.609	0.522	0.609	0.652
		Kappa	0.565	0.534	0.423	0.491	0.614	0.652	0.418	0.678	0.527
Insula	54	BACC	0.972	0.949	0.949	0.961	0.969	0.980	0.885	0.971	0.944
		F1	0.971	0.954	0.954	0.962	0.972	0.981	0.903	0.972	0.941
		Precision	1.000	0.945	0.945	0.981	0.964	0.981	0.864	0.981	1.000
		Sensitivity	0.944	0.963	0.963	0.944	0.981	0.981	0.944	0.963	0.889
		Kappa	0.940	0.899	0.899	0.920	0.940	0.960	0.777	0.940	0.880
M1	28	BACC	0.929	0.950	0.857	0.922	0.939	0.922	0.979	0.922	0.957
		F1	0.923	0.929	0.833	0.906	0.926	0.906	0.949	0.906	0.945
		Precision	1.000	0.929	1.000	0.960	0.962	0.960	0.903	0.960	0.963
		Sensitivity	0.857	0.929	0.714	0.857	0.893	0.857	1.000	0.857	0.929
		Kappa	0.896	0.901	0.783	0.872	0.899	0.872	0.928	0.872	0.925
M2	40	BACC	0.867	0.921	0.896	0.892	0.896	0.921	0.929	0.921	0.942
		F1	0.845	0.909	0.880	0.877	0.880	0.909	0.914	0.909	0.927
		Precision	0.968	0.946	0.943	0.970	0.943	0.946	0.902	0.946	0.905
		Sensitivity	0.750	0.875	0.825	0.800	0.825	0.875	0.925	0.875	0.950
		Kappa	0.762	0.852	0.809	0.807	0.809	0.852	0.855	0.852	0.876
M3	33	BACC	0.894	0.955	0.947	0.955	0.970	0.970	0.970	0.970	0.917
		F1	0.881	0.939	0.937	0.939	0.955	0.955	0.955	0.955	0.903
		Precision	1.000	0.939	0.968	0.939	0.941	0.941	0.941	0.941	0.966
		Sensitivity	0.788	0.939	0.909	0.939	0.970	0.970	0.970	0.970	0.848
		Kappa	0.833	0.910	0.908	0.910	0.933	0.933	0.933	0.933	0.860
M4	29	BACC	0.855	0.951	0.890	0.924	0.890	0.907	0.944	0.907	0.828
		F1	0.824	0.931	0.868	0.909	0.868	0.889	0.915	0.889	0.792
		Precision	0.955	0.931	0.958	0.962	0.958	0.960	0.900	0.960	1.000
		Sensitivity	0.724	0.931	0.793	0.862	0.793	0.828	0.931	0.828	0.655
		Kappa	0.765	0.903	0.821	0.875	0.821	0.848	0.880	0.848	0.730
M5	41	BACC	0.882	0.896	0.876	0.906	0.905	0.893	0.866	0.893	0.888
		F1	0.865	0.874	0.854	0.895	0.889	0.875	0.841	0.875	0.867
		Precision	0.970	0.826	0.854	0.971	0.900	0.897	0.787	0.897	0.857
		Sensitivity	0.780	0.927	0.854	0.829	0.878	0.854	0.902	0.854	0.878
		Kappa	0.787	0.777	0.752	0.831	0.813	0.792	0.717	0.792	0.773
M6	32	BACC	0.915	0.954	0.938	0.954	0.954	0.946	0.923	0.938	0.953
		F1	0.900	0.938	0.921	0.938	0.938	0.935	0.903	0.921	0.951
		Precision	0.964	0.938	0.935	0.938	0.938	0.967	0.933	0.935	1.000
		Sensitivity	0.844	0.938	0.906	0.938	0.938	0.906	0.875	0.906	0.906
		Kappa	0.857	0.908	0.884	0.908	0.908	0.907	0.860	0.884	0.929
Total ASPECTS	100	BACC	0.389	0.400	0.409	0.416	0.504	0.481	0.386	0.513	0.480
		F1	0.491	0.428	0.495	0.568	0.574	0.543	0.423	0.598	0.594
		Precision	0.523	0.522	0.572	0.603	0.612	0.597	0.507	0.626	0.659
		Sensitivity	0.490	0.420	0.490	0.570	0.570	0.530	0.390	0.600	0.580
		Kappa	0.400	0.339	0.397	0.490	0.495	0.454	0.315	0.528	0.510
		Kappa weighted	0.730	0.747	0.770	0.799	0.811	0.790	0.747	0.820	0.796
Total ASPECTS with tolerance	100	BACC	0.748	0.869	0.863	0.842	0.862	0.848	0.839	0.878	0.849
		F1	0.821	0.868	0.906	0.899	0.899	0.903	0.863	0.920	0.887
		Precision	0.835	0.896	0.918	0.912	0.902	0.919	0.892	0.926	0.919
		Sensitivity	0.830	0.860	0.910	0.900	0.900	0.900	0.850	0.920	0.880
		Kappa	0.797	0.836	0.893	0.881	0.882	0.882	0.824	0.905	0.859
		Kappa weighted	0.845	0.890	0.917	0.916	0.921	0.916	0.876	0.931	0.899

**Figure 5:**
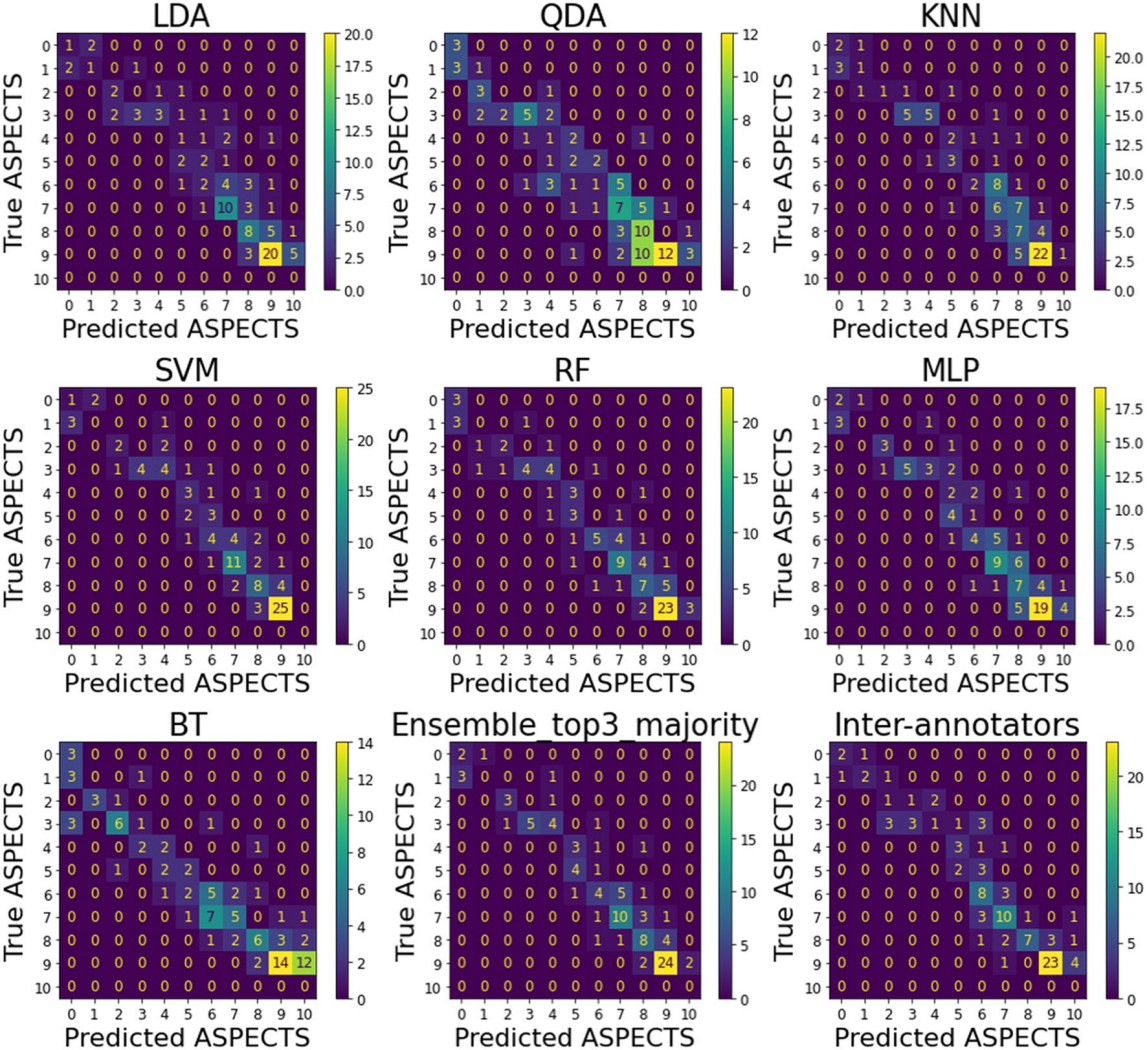
Confusion matrices of ML models in the external testing set (n = 100). A potential perfect model would result in zeros outside the diagonal (i.e., no prediction errors). The cells adjacent to the diagonal represent "acceptable" errors (predicted ASPECTS within ± 1 difference from the ground true ASPECTS).

The accuracy to predict the total ASPECTS was inferior of that to predict regional scores, for both humans and machine. The lowest accuracy can be attributed to the larger number of classes to predict (10 "total ASPECTS" classes, instead of 2 "injury" classes (yes/no) per ROI), and to the imbalance in these classes. As a comparison, as depicted in Supplementary Table 1, the binary ASPECTS of IC have the most imbalanced numbers of classes of all the regions, 71 out of 400 (17.75%). For total ASPECTS, classes 0, 1 and 2 are respectively 8, 15, and 10 samples out of 400 (2%, 3.75%, and 2.5%). Hence, for evaluating total ASPECTS, we favored weighted metrics, such as weighted Cohen’s Kappa coefficient. We also favor the "tolerance" scores, which accept predicted ASPECTS within ± 1 difference from the ground true ASPECTS as true positives. Both weighted and tolerance metrics for total ASPECTS prediction were high and comparable, or superior, to inter-evaluators’ agreement. As shown in the confusion matrices (Fig. 5), although some models have a slight tendency to overestimate ASPECTS (e.g., Quadratic Discriminant Analysis, QDA) while others tend to underestimate (e.g., Linear Discriminant Analysis, LDA) no model had drastic errors. The ensemble model with the 3 best models was again the one with less false predictions beyond the ±1 tolerance in total ASPECTS.

The ASPECTS prediction was more accurate in large infarcts (volume > 14ml, n = 51), compared to small infarcts (volume < 14ml, n = 49) (*p* values in Supplementary Table 4). This is not surprising as large infarcts have large AFV components (i.e., higher percentages of affection per region), and therefore clearer classification features. In addition, the AFV of large infarcts is relatively less affected by inaccuracies in brain mapping. As the infarct volume and location are correlated (i.e., small areas as internal capsule and caudate, which are irrigated by perforating arteries of small caliber, tend to have smaller strokes), large ROIs (such as M1, M2 and M3) have better accuracy performance for all ML models and annotators. There was no significant difference in the prediction accuracy regarding the patient sex (male or female) or race (Black/African America or Caucasian), time from stroke onset (> or < 6 h), magnetic field (1.5 T or 3 T), and infarct side (left or right).

### Prediction interpretability

**Figure 6:**
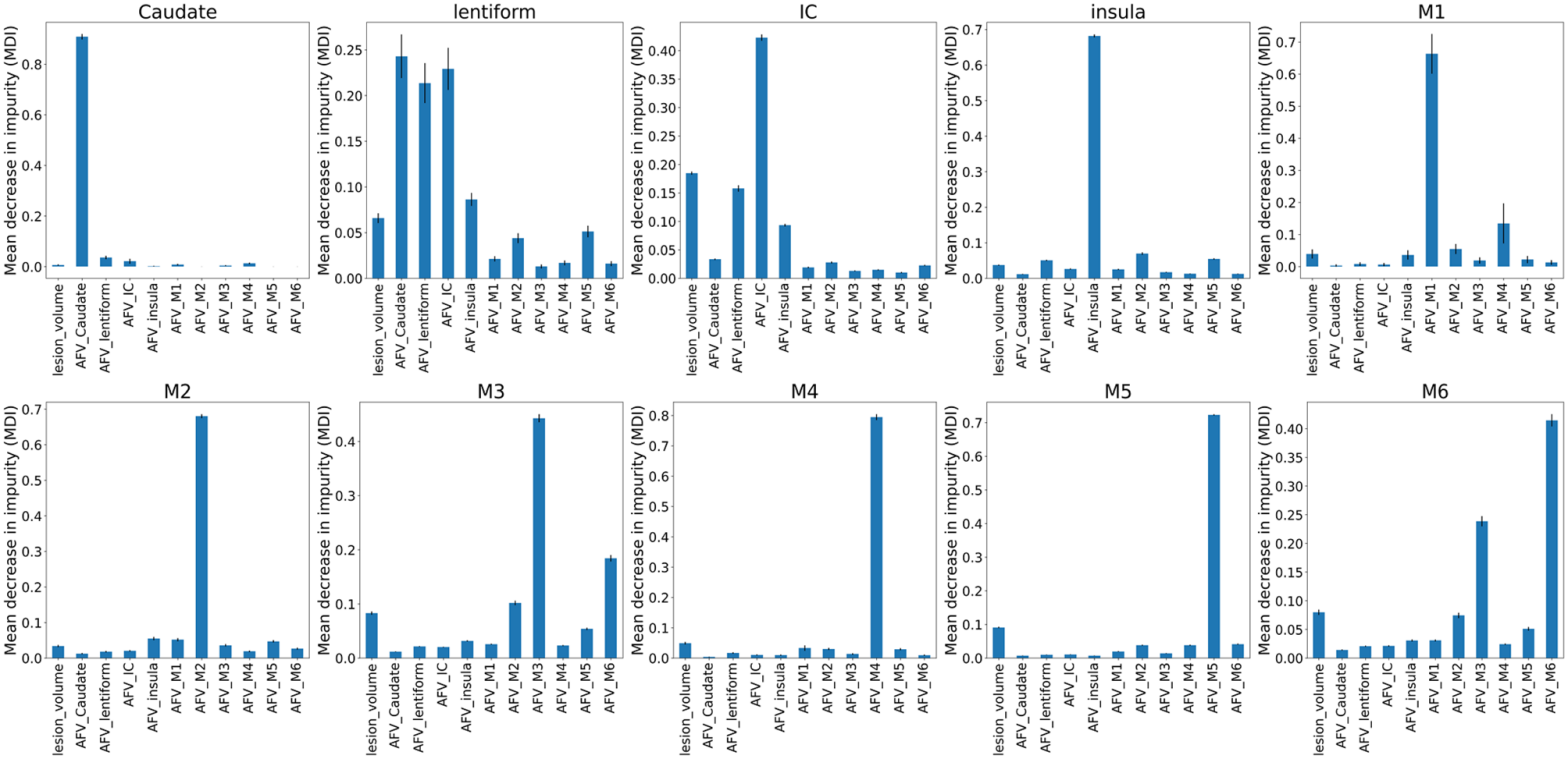
Feature importance, as revealed by the Mean Decrease in Impurity (MDI) of the Random Forest (RF) models. The MDI is proportional to the importance of the features (the AFVs and lesion volume, in the x-axis) to predict the injury of the region in question (title of each graph). The AFVs represent the proportion of each ROI affected by the infarct. Note that the dominant AFV component agrees with the prediction of injury in the corresponding region and is followed by the AFV component of its spatially neighboring regions.

Instead of building black-box ML models, we aimed to provide interpretable models to elucidate whether the machine uses features of biological relevance, similar to humans. Fig. 6 and Supplementary Table 5 indicate the importance of features in the RF models. In general, the most important feature was the percentage of injury in the region in question, followed by the injury of neighbor regions, as shown in Fig. 6. For example, the top 3 features for each ROI are mostly similar to the top correlations of visual ASPECTS and AFV (Table 2), indicating that, in general, RF models and humans are using very similar features for scoring. The permutation feature importance test (Supplementary Materials) showed feature selection very similar to that from the impurity decrease method and demonstrated consistency of the importance features learned in the training set, and their generalization to the testing set.

**Figure 7:**
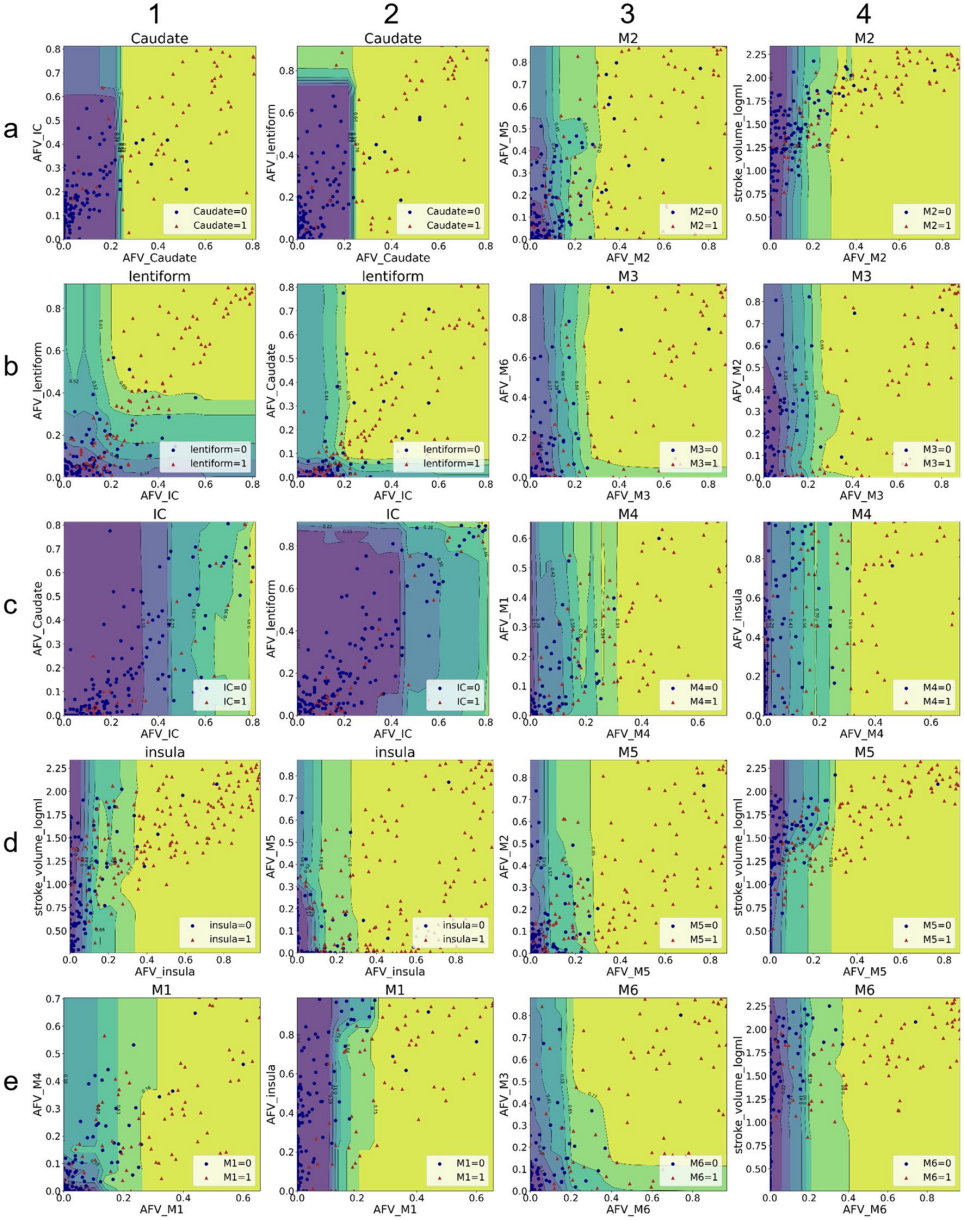
Partial dependent plots (PDPs) showing the top 3 features of Random Forest (RF) models for each ROI. The PDPs indicate the average marginal effect of the AFV on classifying each ROI (title of each graph) in infarcted (1) or not infarcted (0). As the three most important features are shown, each region is represented by a pair of graphics in which the x-axis shows the most important classification feature and the y-axis shows the second and third, respectively. The colors show the topology of the probabilistic classification. For example, top left graphic shows that caudate usually scores "1" (i.e., infarcted) if above 25% of its volume is occupied by the infarct mask (red dots in the yellow area). However, even if caudate injury is below this level, a case may score "1" if the neighboring lentiform is highly affected (> 80%; red dots in the green area). This indicates that, similarly to humans, AI uses a more complex classification approach than the single binary threshold of injury for the region in question to determine injury.

The Partial Dependence Plots (PDP) in Fig. 7 show that the predicted ROI ASPECTS primarily depends on the degree of injury of the ROI in question, as expected. However, just as happened with the human scoring, there are "joined" conditions, in which the affection of one region influences the classification of another region. Distinct scenarios were observed:

1. In two neighboring regions, highly correlated in the visual analysis (e.g., caudate and lentiform), a given ROI, even minimally affected, may score 1 if its neighbor is highly affected. For example, panel a2 of Fig. 7 shows that caudate scores 1 when AFV-Caudate > 0.25 (i.e., if the infarct core affects more than 25% of caudate). However, if AFV-lentiform > 0.8, caudate still scores 1 even if AFV-caudate < 0.25. We again note that possible imprecision in the brain registration caused by midline shifts and / or hydrocephalus can be partially responsible for such observations in these specific mesial and periventricular structures.
2. The classification of several labels substantially (although not predominantly) depends on "joined" conditions. For example, lentiform = 1 (i.e., "injured") hardly depends on a single threshold; RF considers AFV-IC and AFV-lentiform higher than 0.2 and 0.3, or AFV-IC and AFV-Caudate higher than 0.2 and 0.1, respectively, to classify lentiform as affected, as shown in Fig. 7 panels b1 and b2.
3. In some "cortical" segments (M1-M6 and insula) a high AFV of adjacent regions may have a slightly negative effect on ASPECTS. For example, the panel e2 of Fig. 7 shows that when AFV-insula is too high (> 0.8), AFV-M1 has to be higher than the threshold used when M1 is sole injured, for M1 be classified as infarcted.
4. It is hard to account for how each pair of AFV features affects IC classification. The PDP illustrates the explanation of two features at most (via marginal expectation of other features). Because the RF model could depend on more than 2 features to predict the ASPECTS IC, the PDP does not provide any suitable interpretation for IC classification in RF models.

In general, the PDP showed that the ASPECTS prediction in large ROIs, such as M1, M2, M3, dominantly depends on the ROI AFV. In these regions, the simple "binary threshold method" (BT) resulted in similar performance to those of more complex methods as RF (Table 3). On the other hand, small ROIs with registration issue, ambiguous anatomical boundaries, or less incident in our sample (for example, IC, lentiform or caudate), need multiple features for classification. In these cases, the BT showed inferior performance to RF and others, as BT does not consider the joined conditioning by definition.

While the methods above expose the features implied in the classification at group level, it is important to expose the features used for the ASPECTS ranking of each individual. The "SHapley Additive exPlanations" (SHAP)^15^ were adapted in our pipeline along with our complete training set to explain the reasoning behind the model’s ASPECTS prediction for any given new sample. Supplementary Fig. 3 illustrates how the pre-trained RF model in ADS^13^ interprets the contribution of each AFV component to predict ASPECTS in a given region.

## Discussion

We created a fully automated system to calculate ASPECTS, with accuracy comparable to that inter- evaluator, robust to major technical, lesion, and population variations. The agreement of the final ensemble model with the consensual visual ASPECTS was virtually perfect for most of the regions. Among BACC of all methods, including inter-annotators, the lowest agreement was in the internal capsule (IC). We note that IC offers extra challenges for both humans and machine by its anatomical nature: in addition to be a “small strip” with ill-defined axial in-plane boundaries in the low-resolution clinical DWI, evaluators tend to disagree on its rostral-caudal limits and might inconsistently consider its involvement as an extension of neighboring caudate or lenticular infarcts. The second lowest agreement for the automated methods was found in caudate, although the agreement with the ground truth ASPECTS was still very acceptable (BACC=0.850 for the RF model, BACC = 0.832 for the ensemble model). This can be partially attributed to issues of brain mapping affecting primarily the mesial and periventricular regions, which may occur in populations with common midline shift and hydrocephalus. To ameliorate this issue, our system offers the option to recalculate ASPECTS using a non-linear mapping. As shown in Fig. 4, this recalculation provides a more accurate quantification of the infarct in periventricular structures and, consequently, more accurate ASPECTS prediction, at the cost of increasing the time for image processing in about 3 minutes.

The bivariate and the feature analyses revealed that, as expected, the main feature determining the ASPECTS in a given region is the percentage of the respective region affected by the infarct. However, other additional features were used by both humans and machine. The analysis of human visual ASPECTS demonstrated high correlation between the scores of areas in which infarcts tend to coalesce, and adjacent ROIs. The feature analysis of RF and other ML models revealed that the global lesion pattern, or the infarction of spatially adjacent regions, influences the automated classification in a very similar way as it affects the human classification. For example, Fig. 6 and Table 2 show that the computational models and the humans tend to score lentiform as injured if they find that the neighbors IC and caudate are also injured. Inversely, they tend to minimize injuries in large cortical areas (M1-M3) when a neighboring large cortical area is severely damaged, apparently attributing the supposed injury to a "spread" of the infarct rather than a primary infarct on that specific area. Finally, in regions of more challenging visual analysis, the agreement with human evaluation depends on the combination of injury in different areas. Aligned with this fact, the performance of the simple binary threshold method in these areas (e.g., lentiform, IC, insula) is inferior to that of other more complex models. Interestingly, these areas are those mostly inter-correlated in the visual analysis, supporting the idea that machine and humans are considering similar features.

The feature analysis enriches the AI models, increasing their interpretability and their potential usefulness. Therefore, our system ADS^13^ is suited to output not only the predicted ASPECTS but also the feature vector (AFV) showing the proportion of each brain region affected by the infarct (as in Figs. 3 and 4), the graphic representation of how the pre-trained model interprets the AFV components to predict scores in each region (Supplementary Fig. 3), as well as the probabilities of such ASPECTS predictions (as in Figs. 3 and 4). This information can be used as an indirect validation of the automated scores, or a metric of certainty about them, as well as for other quantitative proposes. Another particularity is that our system is flexible to different brain parcellation schemes. Therefore, different ROIs can be easily adopted, either to test their clinical significance, or to provide better metrics when their relevance is established^16^. Finally, our system is completely automated, including the lesion segmentation^17^. We note that because the automated and manual lesion segmentation do not perfectly agree^17^, the models currently available in ADS were retrained with the automated segmented lesions (accuracy summarized in Supplementary Tables 6, 7 and 8). The accuracy of the models trained with manual lesion segmentation or with automated lesion segmentation by ADS were virtually the same, indicating that these latter are suited for a complete automated pipeline for stroke imaging processing, that includes infarct segmentation.

In summary, using the original DWI as input, we created a fully automated system that outputs ASPECTS, in addition to the previously reported^17^ 3D digital stroke mask, volume, and the feature vector of anatomical regions affected by the acute stroke. This system is publicly available, runs in real time, in local computers, with minimal computational requirements, and it is readily useful for non-expert users. The addition of an efficient ASPECTS calculation indicates that ADS is able to extract personalized information of potential clinical relevance from clinical MRIs of patients with acute strokes.

## Methods

### Image processing

This study included Magnetic Resonance Images (MRIs) of patients admitted to the Johns Hopkins Stroke Center with the clinical diagnosis of acute stroke, between 2009 and 2019. This dataset is public^18^. All methods of this study were carried out in accordance with relevant guidelines and regulations (IRB00290649, IRB00228775). We included baseline MRIs adequate for clinical analysis with evidence of ischemic stroke in the diffusion weighted images (DWI), as in our previous study on automated lesion segmentation^17^. Herein, we included infarcts affecting exclusively the territory of the middle cerebral artery (MCA) with non-ten ASPECTS score (n = 400).

MRIs were obtained on eleven scanners from four different vendors, two magnetic fields (1.5 T and 3 T), with dozens of different protocols. The DWIs had high in plane (axial) resolution (1.2 × 1.2 mm^2^, or less), and typical clinical large slice thickness (ranging from 3 to 6 mm). The delineation of the ischemic core was defined in the DWI by two experienced evaluators and revised by a neuroradiologist until reaching a final decision by consensus (details in^19^). The human segmentation is here considered the “ground true". The “automated” lesion segmentation was performed with ADS, according to^17^. The DWIs were mapped to a common template in MNI space (JHU_MNI^20^) by 12-parameter linear transformation; the transformation matrix was then applied to the binary stroke masks. Details about the mapping, including used parameters and quality control, are in our publications describing the dataset^19^ and the lesion segmentation algorithm^17^.

### Visual ASPECTS

An ASPECTS atlas (Fig. 2) was created using the JHU_SS_MNI template^20^, by selecting regions of interest (ROIs) from our previously published atlas^21–23^. The ASPECTS atlas defines the 10 areas considered in the ASPECTS system: the caudate, the lentiform, the internal capsule (IC), the insula, and the cortical / subcortical regions from M1-M6^24^. This proposed ASPECTS deformable 3D atlas is publicly available in ADS^13^. The visual ASPECTS rating was done by two evaluators, and finally defined by consensus with a neuroradiologist. The evaluation was done on the DWI and ADC images in MNI space, having access to the overlapped ASPECTS map. Raters used the typical clinical scoring system (1 if the given region is considered affected by the infarct, 0 if not. For the total ASPECTS, each point was subtracted from 10, which is the normal). The consensus visual ASPECTS are considered as "ground truth" scores in this study. The frequency of ASPECTS per score classes and per region is summarized in Supplementary Table 1.

### ASPECTS feature vectors (AFV)

We used the percentages of ASPECTS ROIs affected by the infarct as the feature vector of our classification models. In each ROI, this percentage is the number of ROI voxels where the stroke mask = 1 divided by the total number of bilateral ROI voxels (Fig. 3). Left and right sides are combined by summation. Infarct volume (in *log*10 ml) was also included into the feature vector as it correlates to infarct location. In total, AFV has 11 features: the percentage of infarct in each of the 10 ASPECTS ROIs plus the infarct volume. In this study, ML prediction models used AFVs derived from the manual segmentation of the infarcts and their results are shown in the main manuscript. The results of models trained with the AFV derived from automated segmentation of the infarcts^17^ are summarized in the supplementary material. The models and parameters are public in ADS^13^.

### Machine learning (ML) classification models to predict ASPECTS

We developed, validated, and tested seven models (described below) to predict the consensus visual classification (injured = 1, not-injured = 0) in each of the 10 ASPECTS ROIs, using the AFVs. All ML models were 5-fold cross validated over the training set (300 subjects, 75%) for searching hyperparameters and tested in external 100 subjects (flowchart in Fig. 1). The models’ hyperparameters with the top performances (BACC and F1 score) from the first-run 5-fold cross validation were further selected via 100 repeat 5-fold cross validation. The parameter searching sets, final optimal parameters, and cross validation results are in Supplementary Tables 2 and 3.

The simplest model, the Binary Threshold (BT), was built to classify visual ASPECTS via thresholding its corresponding ROI component in the AFV for each subject. The threshold can be interpreted as the minimum percentage of the ROI that has to be affected by the infarct to lead its classification as injured ROI. The threshold for each ROI was the minimal level to achieve the highest sum of BACC and F1 score, found by cross-validation in the training set. The optimal thresholds for each ROI are summarized in Supplementary Table 2.

The remaining six models, Linear Discriminant Analysis (LDA), Quadratic Discriminant Analysis (QDA), Random Forest (RF), K-nearest Neighbors (KNN), Support Vector Machine (SVM), Multi-layer Perceptron (MLP), were implemented via scikit-learning module^25^. Two ensemble models were also tested, one using all the models and the other with the best three models (SVM, RF, and MLP). The ensembles used majority voting policy. The top models were chosen according to the average performance (BACC, F1) of 100 repeated 5-fold cross validation among 300 training samples. Because the ensemble model that combined all the models had performance slightly inferior to that of the ensemble of the three best models, only the results of the latter are shown in Table 3.

### Feature analysis

We explored how ASPECTS attributed to different ROIs relate to each other, as well as the relationship between visual ASPECTS and AFV for each ROI, and between different AFV components, using correlation coefficients (Table 2). We used different methods to identify the important features selected in successful ML models to predict ASPECTS, and implemented a system to expose these features, comprehensively, in a given new sample.

The analysis of feature importance aims to inspect how annotators and ML models use the AFVs to attribute ASPECTS. The analyses presented here are based on RF models, which had the best average performance (BACC, F1) among all ML models. The impurity-based feature importance analysis^26,27^ was conducted using the RF models 100 times simulated on the training set. The Mean Decrease in Impurity (MDI), shown in Fig. 6, indicates the feature importance (high MDIs correspond to the most important features). MDI describes the weighted mean of RF’s improvement in Gini-gain splitting criterion produced by each feature variable. We also conducted a permutation RF feature importance test^28^ (100 interactions) via BACC, using the training and testing set separately (Supplementary Figs. 1 and 2), to illustrate the consistency in feature learning and their potential generalization, respectively.

The Partial Dependence Plots (PDP)^29,30^ were used to provide an intuitive global interpretation of how selected features affect the models’ prediction. The PDP, shown in Fig. 7, capture the average marginal effect on predictions for selected features via marginalizing out all other features. PDP gives global model explanations over the testing set. The SHapley Additive exPlanations (SHAP)^15^ was included in the ADS pipeline, to generate intuitively comprehensible graphical explanations of predicted ASPECTS in a new given sample. SHAP computes the Shapley values^31^ of features via coalitional game theory to indicate how to fairly distribute prediction of an instance among features. Because Shapley feature value is linearly additive, this value can be directly added or subtracted from the probability of predicts, making the models’ interpretation straightforward.

### Measures of model performance

Denote True Positives, False Positives, True Negatives, and False Negatives, as TP, FP, TN, and FN, respectively. We evaluated:

1. Balanced accuracy (BACC)^32^: to avoid performance inflation resulted from imbalanced classes, BACC is used instead of accuracy.
  1 $${\text{Balanced}}\,{\text{Accuracy}}\,{\text{(BACC) = }}\frac{1}{2}\left( {\frac{{{\text{TP}}}}{{{\text{TP}} + {\text{FP}}}} + \frac{{{\text{TP}}}}{{{\text{TP}} + {\text{FN}}}}} \right)$$
2. Precision: a metric to evaluate how accurate a model’s positive predict is true.
  2 $${\text{Precision}} = \frac{{{\text{TP}}}}{{{\text{TP}} + {\text{FP}}}}$$
3. Sensitivity: a metric to evaluate how the model’s ability to detect the positive cases among dataset.
  3 $${\text{Sensitivity}} = \frac{{{\text{TP}}}}{{{\text{TP}} + {\text{FN}}}}$$
4. F1 score: the harmonic mean of the precision and sensitivity.
  4 $${\text{F1}}\,{\text{Score}} = \frac{2}{{{\text{Sensitivity}}^{ - 1} + {\text{Precision}}^{ - 1} }}$$
5. Cohen’s Kappa coefficient^33^, *κ*: a statistic to measure the agreement between annotators. Besides evaluating our inter-annotator performance, we also like to consider ML models as extra annotators and evaluate their performance in the test set, completely hold-out from training.
  $$\kappa = \frac{{p_{o} - p_{e} }}{{1 - p_{e} }}$$
  where *po* is the empirical probability of agreement among raters, and *pe* is the expected agreement between random raters. *pe* is estimated via the empirical prior probability of each class of raters.

The metrics to evaluate binary classification defined as above apply to each ASPECTS ROI (which can be either 0 or 1). For the total ASPECTS (ten minus the summation of ASPECTS from each ROI in an individual), we calculated the metrics for each of the 10 classes and the weighted average of all classes. The weights are supported by the number of samples of each class. In addition, Cohen’s Kappa score was calculated by "no weighted", or linear weighted average across multi-classes. As for practical applications, a predicted total ASPECTS within ± 1 difference from the ground true ASPECTS is acceptable, we additionally computed all the above metrics using this tolerance margin. Therefore, the denoted "tolerance" indices consider a predicted ASPECTS within ± 1 difference from the ground true ASPECTS as a true positive.

### System implementation

The statistical significance testing was performed by ANOVA test in module "bioinfokit" for continuous data, and by Chi-square test via chi2_contingency module in scipy for categorical data. The metrics of model performance were implemented by scikit-learning module. All the evaluated methods and models were built with TensorFlow^34^ (tensorflow-gpu version is 2.0.0) and Keras^35^ (2.3.1) framework on Python 3.6 Imaging processing and analysis were built with Nibabel^36^, Scipy^37^, Dipy^38^ and Scikit-learning^25^, Scikit-image^25,39^, SHAP^15^. The experiments run on a machine with an Intel Core (Intel(R) Xeon(R) CPU E5-2620 v4 @ 2.10GHz) with 2 NVIDIA TITAN XP GPUs (with CUDA 10.1).

## Supplementary Information

Supplementary Information.

## Data Availability

The tool developed here for automated calculation of ASPECTS is available at NITRC^13^. The dataset that used for the development is available at ICPSR^18^and can be freely downloaded after registration and signing of Data Use Agreement.
