# Supplementary material for: Automatic comprehensive aspects reports in clinical acute stroke MRIs
Source: Sci Rep. 2023 Mar 7;13:3784. doi: 10.1038/s41598-023-30242-6 (PMC9992659; doi:10.1038/s41598-023-30242-6)
Supplement: Supplementary file 1 — Supplementary Information. [file 41598_2023_30242_MOESM1_ESM.pdf]

AUTOMATIC COMPREHENSIVE ASPECTS REPORTS IN CLINICAL ACUTE STROKE MRIs: Supplementary Information

SUPPLEMENTARY TABLES

Dataset	Total	Training	Testing	p-value
ASPECTS	6.50(2.42); 7[5,8.25]	6.58(2.32); 7[5,8]	6.4(2.59); 7[5,9]	0.238
Total ASPECTS				
9	100	72	28	
8	69	55	14	
7	80	65	15	
6	37	26	11	
5	33	28	5	0.305
4	21	16	5	
3	27	16	11	
2	10	6	4	
1	15	11	4	
0	8	5	3	
ROI ASPECTS				0.483
Caudate*	108	72	36	0.027
lentiform*	187	128	59	0.007
IC	72	49	23	0.176
insula	228	174	54	0.56
M1	111	83	28	0.949
M2	147	107	40	0.51
M3	126	93	33	0.804
M4	119	90	29	0.95
M5	178	137	41	0.486
M6	125	93	32	0.95

Supplementary Table 1. Distribution of total ASPECTS (top) and binary ASPECTS per region of interest, ROI. The numbers are the counts of cases that scored 1 (i.e., injury) in the regions in question. Statistical significant differences in distributions between testing and training sets are marked with "*". The testing set has relatively more cases of infarct in caudate and lentiform than the training set.

BT		
param_grid	Best model parameters	
{'th':range(0,1,0.01)}	Caudate	{'th': 0.23925200000000005}
	lentiform	{'th': 0.08901999999999999}
	IC	{'th': 0.4826439999999999}
	insula	{'th': 0.127018}
	M1	{'th': 0.10889000000000001}
	M2	{'th': 0.159972}
	M3	{'th': 0.10973799999999999}
	M4	{'th': 0.11511400000000001}
	M5	{'th': 0.06666200000000001}
	M6	{'th': 0.16876600000000003}
RF		
param_grid	Best model parameters	
{'criterion' : ['gini'],<br>'max_features' : ['sqrt', None],<br>'max_depth': [None,2,4,6,8,10],<br>'n_estimators' :[10,25,50,100,250,500,750,1000]}	Caudate	{'criterion': 'gini', 'max_depth': 2, 'max_features': None, 'n_estimators': 50}
	lentiform	{'criterion': 'gini', 'max_depth': 6, 'max_features': 'sqrt', 'n_estimators': 100}
	IC	{'criterion': 'gini', 'max_depth': 4, 'max_features': None, 'n_estimators': 1000}
	insula	{'criterion': 'gini', 'max_depth': 6, 'max_features': None, 'n_estimators': 500}
	M1	{'criterion': 'gini', 'max_depth': 4, 'max_features': None, 'n_estimators': 10}
	M2	{'criterion': 'gini', 'max_depth': 6, 'max_features': None, 'n_estimators': 100}
	M3	{'criterion': 'gini', 'max_depth': 10, 'max_features': 'sqrt', 'n_estimators': 1000}
	M4	{'criterion': 'gini', 'max_depth': 4, 'max_features': None, 'n_estimators': 100}
	M5	{'criterion': 'gini', 'max_depth': 6, 'max_features': None, 'n_estimators': 500}
	M6	{'criterion': 'gini', 'max_depth': 8, 'max_features': 'sqrt', 'n_estimators': 500}
KNN		
param_grid	Best model parameters	
{'n_neighbors':[2,3,4,5,6,7,8,9,10,11,12,13,14,15,16,17,18,19,20,<br>25,30,35,40,45,50,60,70,80,90,100,150,200],<br>'weights' : ['uniform', 'distance']}	Caudate	{'n_neighbors': 6, 'weights': 'distance'}
	lentiform	{'n_neighbors': 7, 'weights': 'distance'}
	IC	{'n_neighbors': 6, 'weights': 'distance'}
	insula	{'n_neighbors': 14, 'weights': 'distance'}
	M1	{'n_neighbors': 10, 'weights': 'distance'}
	M2	{'n_neighbors': 4, 'weights': 'distance'}
	M3	{'n_neighbors': 5, 'weights': 'distance'}
	M4	{'n_neighbors': 8, 'weights': 'distance'}
	M5	{'n_neighbors': 10, 'weights': 'distance'}
	M6	{'n_neighbors': 3, 'weights': 'uniform'}
SVM		
param_grid	Best model parameters	
{'kernel': ['rbf'],<br>'gamma': [1e-3, 1e-4, 1e-5],<br>'C': [1,2,3,4,5,6,7,8,9,10,15,20,25,30, 50, 75, 100, 200, 300, 500]},<br>{'kernel': ['linear'],<br>'C': [1,2,3,4,5,6,7,8,9,10,15,20,25,30, 50, 75, 100, 200, 300, 500]}	Caudate	{'C': 50, 'kernel': 'linear'}
	lentiform	{'C': 300, 'gamma': 0.001, 'kernel': 'rbf'}
	IC	{'C': 25, 'kernel': 'linear'}
	insula	{'C': 300, 'kernel': 'linear'}
	M1	{'C': 200, 'kernel': 'linear'}
	M2	{'C': 15, 'kernel': 'linear'}
	M3	{'C': 30, 'kernel': 'linear'}
	M4	{'C': 3, 'kernel': 'linear'}
	M5	{'C': 100, 'kernel': 'linear'}
	M6	{'C': 300, 'kernel': 'linear'}
MLP		
param_grid	Best model parameters	
{'hidden_layer_sizes' : [64,128,256,512,1024],<br>'alpha': [1E-5, 1E-4, 1E-3],<br>'max_iter' : [200,500,800],<br>'learning_rate_init' : [1E-4,1E-3]}	Caudate	{'alpha': 0.0001, 'hidden_layer_sizes': 1024, 'learning_rate_init': 0.001, 'max_iter': 500}
	lentiform	{'alpha': 1e-05, 'hidden_layer_sizes': 1024, 'learning_rate_init': 0.001, 'max_iter': 200}
	IC	{'alpha': 1e-05, 'hidden_layer_sizes': 1024, 'learning_rate_init': 0.001, 'max_iter': 500}
	insula	{'alpha': 1e-05, 'hidden_layer_sizes': 1024, 'learning_rate_init': 0.001, 'max_iter': 800}
	M1	{'alpha': 0.0001, 'hidden_layer_sizes': 256, 'learning_rate_init': 0.001, 'max_iter': 500}
	M2	{'alpha': 1e-05, 'hidden_layer_sizes': 256, 'learning_rate_init': 0.001, 'max_iter': 800}
	M3	{'alpha': 0.0001, 'hidden_layer_sizes': 512, 'learning_rate_init': 0.001, 'max_iter': 800}
	M4	{'alpha': 1e-05, 'hidden_layer_sizes': 1024, 'learning_rate_init': 0.001, 'max_iter': 200}
	M5	{'alpha': 0.001, 'hidden_layer_sizes': 1024, 'learning_rate_init': 0.001, 'max_iter': 800}
	M6	{'alpha': 0.0001, 'hidden_layer_sizes': 128, 'learning_rate_init': 0.001, 'max_iter': 500}

Supplementary Table 2. Table of hyperparameters for the ML models for ASPECTS prediction

ROI	Number of occurrences	Metric	LDA	QDA	KNN	SVM	RF	MLP	BT	Ensemble all majority	Ensemble top5 majority	Ensemble top3 majority
Caudate	72	BACC	0.880	0.922	0.873	0.889	0.932	0.893	0.938	0.938	0.938	0.938
		F1	0.835	0.862	0.821	0.842	0.894	0.844	0.897	0.909	0.909	0.921
		Precision	0.893	0.826	0.878	0.883	0.892	0.875	0.883	0.915	0.915	0.955
		Sensitivity	0.792	0.907	0.782	0.814	0.901	0.826	0.917	0.903	0.903	0.889
lentiform	128	BACC	0.887	0.879	0.901	0.900	0.894	0.895	0.816	0.934	0.920	0.932
		F1	0.869	0.861	0.886	0.884	0.877	0.879	0.792	0.923	0.908	0.920
		Precision	0.868	0.835	0.858	0.841	0.846	0.859	0.761	0.909	0.894	0.896
		Sensitivity	0.876	0.893	0.920	0.935	0.915	0.904	0.835	0.938	0.922	0.945
IC	49	BACC	0.665	0.699	0.658	0.631	0.702	0.715	0.717	0.792	0.792	0.790
		F1	0.454	0.469	0.440	0.389	0.535	0.545	0.517	0.698	0.698	0.716
		Precision	0.574	0.416	0.595	0.588	0.747	0.675	0.522	0.811	0.811	0.906
		Sensitivity	0.391	0.557	0.370	0.307	0.438	0.482	0.535	0.612	0.612	0.592
insula	174	BACC	0.885	0.856	0.854	0.885	0.879	0.891	0.861	0.940	0.936	0.977
		F1	0.888	0.873	0.875	0.896	0.902	0.906	0.887	0.944	0.941	0.980
		Precision	0.944	0.896	0.885	0.922	0.891	0.916	0.880	0.970	0.964	0.983
		Sensitivity	0.840	0.853	0.868	0.875	0.917	0.899	0.897	0.920	0.920	0.977
M1	83	BACC	0.851	0.882	0.829	0.895	0.883	0.892	0.895	0.940	0.938	0.932
		F1	0.809	0.809	0.761	0.854	0.827	0.850	0.821	0.920	0.915	0.908
		Precision	0.927	0.761	0.809	0.885	0.823	0.882	0.758	0.938	0.926	0.925
		Sensitivity	0.725	0.873	0.727	0.832	0.839	0.829	0.904	0.904	0.904	0.892
M2	107	BACC	0.855	0.849	0.821	0.863	0.865	0.863	0.864	0.921	0.919	0.938
		F1	0.820	0.803	0.770	0.828	0.827	0.827	0.819	0.904	0.900	0.924
		Precision	0.908	0.780	0.808	0.885	0.843	0.867	0.776	0.931	0.922	0.942
		Sensitivity	0.754	0.833	0.744	0.784	0.818	0.796	0.872	0.879	0.879	0.907
M3	93	BACC	0.868	0.899	0.845	0.926	0.928	0.924	0.929	0.966	0.963	0.966
		F1	0.839	0.851	0.801	0.907	0.902	0.901	0.884	0.957	0.951	0.957
		Precision	0.965	0.823	0.903	0.948	0.909	0.926	0.838	0.967	0.957	0.967
		Sensitivity	0.748	0.889	0.727	0.875	0.900	0.882	0.944	0.946	0.946	0.946
M4	90	BACC	0.847	0.858	0.829	0.888	0.892	0.882	0.881	0.921	0.914	0.919
		F1	0.802	0.791	0.765	0.851	0.848	0.842	0.823	0.902	0.886	0.897
		Precision	0.911	0.759	0.800	0.890	0.856	0.882	0.800	0.940	0.907	0.929
		Sensitivity	0.726	0.835	0.741	0.822	0.848	0.815	0.860	0.867	0.867	0.867
M5	137	BACC	0.879	0.872	0.870	0.933	0.930	0.929	0.869	0.968	0.965	0.997
		F1	0.862	0.861	0.857	0.927	0.924	0.923	0.861	0.966	0.963	0.996
		Precision	0.970	0.859	0.873	0.968	0.924	0.947	0.809	0.992	0.985	0.993
		Sensitivity	0.780	0.869	0.846	0.892	0.927	0.902	0.926	0.942	0.942	1.000
M6	93	BACC	0.883	0.910	0.912	0.937	0.925	0.928	0.909	0.942	0.947	0.953
		F1	0.861	0.868	0.887	0.917	0.901	0.907	0.873	0.928	0.934	0.940
		Precision	0.978	0.844	0.931	0.935	0.919	0.938	0.879	0.955	0.955	0.956
		Sensitivity	0.774	0.898	0.852	0.903	0.887	0.883	0.876	0.903	0.914	0.925
Average of ROIs		BACC	0.850	0.863	0.839	0.875	0.883	0.881	0.868	0.926	0.923	0.934
		F1	0.804	0.805	0.786	0.830	0.844	0.842	0.818	0.905	0.900	0.916
		Precision	0.894	0.780	0.834	0.875	0.865	0.877	0.791	0.933	0.923	0.945
		Sensitivity	0.741	0.841	0.758	0.804	0.839	0.822	0.857	0.881	0.881	0.894

Supplementary Table 3. Performance of the ML models according to cross validation on the training set

Model	Group	ROI	N_occurrences	BACC	F1	Precision	Sensitivity	Kappa	Kappa weighted
RF	Large	Caudate	23	0.863	0.851	0.833	0.870	0.724	0.724
		lentiform	29	0.943	0.947	0.964	0.931	0.881	0.881
		IC	10	0.838	0.778	0.875	0.700	0.731	0.731
		insula	44	0.917	0.977	0.977	0.977	0.834	0.834
		M1	26	0.942	0.939	1.000	0.885	0.883	0.883
		M2	38	0.844	0.889	0.941	0.842	0.625	0.625
		M3	30	0.976	0.984	0.968	1.000	0.959	0.959
		M4	26	0.922	0.920	0.958	0.885	0.843	0.843
		M5	33	0.843	0.896	0.882	0.909	0.696	0.696
		M6	29	0.943	0.947	0.964	0.931	0.881	0.881
		Total ASPECTS	51	0.542	0.532	0.601	0.529	0.465	0.758
		Total ASPECTS with tolerance	51	0.863	0.865	0.877	0.863	0.841	0.874
	Small	Caudate	13	0.794	0.727	0.889	0.615	0.652	0.652
		lentiform	30	0.924	0.931	0.964	0.900	0.831	0.831
		IC	13	0.678	0.526	0.833	0.385	0.431	0.431
		insula	10	0.987	0.952	0.909	1.000	0.939	0.939
		M1	2	0.750	0.667	1.000	0.500	0.657	0.657
		M2	2	0.989	0.800	0.667	1.000	0.790	0.790
		M3	3	0.822	0.667	0.667	0.667	0.645	0.645
		M4	3	1.000	1.000	1.000	1.000	1.000	1.000
		M5	8	0.875	0.857	1.000	0.750	0.834	0.834
		M6	3	0.822	0.667	0.667	0.667	0.645	0.645
		Total ASPECTS	49	0.393	0.612	0.646	0.592	0.360	0.514
		Total ASPECTS with tolerance	49	0.674	0.897	0.897	0.898	0.830	0.797
Ensemble	Large	Caudate	23	0.881	0.870	0.870	0.870	0.762	0.762
		lentiform	29	0.977	0.983	0.967	1.000	0.960	0.960
		IC	10	0.876	0.800	0.800	0.800	0.751	0.751
		insula	44	0.906	0.966	0.977	0.955	0.766	0.766
		M1	26	0.922	0.920	0.958	0.885	0.843	0.843
		M2	38	0.883	0.901	0.970	0.842	0.679	0.679
		M3	30	0.976	0.984	0.968	1.000	0.959	0.959
		M4	26	0.942	0.941	0.960	0.923	0.882	0.882
		M5	33	0.843	0.896	0.882	0.909	0.696	0.696
		M6	29	0.943	0.947	0.964	0.931	0.881	0.881
		Total ASPECTS	51	0.520	0.497	0.536	0.510	0.441	0.780
		Total ASPECTS with tolerance	51	0.913	0.901	0.910	0.902	0.887	0.918
	Small	Caudate	13	0.794	0.727	0.889	0.615	0.652	0.652
		lentiform	30	0.914	0.933	0.933	0.933	0.828	0.828
		IC	13	0.692	0.556	1.000	0.385	0.479	0.479
		insula	10	0.987	0.952	0.909	1.000	0.939	0.939
		M1	2	1.000	1.000	1.000	1.000	1.000	1.000
		M2	2	1.000	1.000	1.000	1.000	1.000	1.000
		M3	3	0.822	0.667	0.667	0.667	0.645	0.645
		M4	3	0.667	0.500	1.000	0.333	0.484	0.484
		M5	8	0.875	0.857	1.000	0.750	0.834	0.834
		M6	3	0.822	0.667	0.667	0.667	0.645	0.645
		Total ASPECTS	49	0.418	0.648	0.703	0.633	0.410	0.552
		Total ASPECTS with tolerance	49	0.710	0.928	0.925	0.939	0.896	0.849

Supplementary Table 4. Performance of Random Forest (RF) and Ensemble models on the testing set, according to the infarct volume. "Large" includes cases with infarct core > 14ml; "Small" are infarct core < 14ml. Although still acceptable, the performance decreases in "small" infarcts.

	ROI	stroke volume log ml	AFV_Caudate	AFV_lentiform	AFV_IC	AFV_insula	AFV_M1	AFV_M2	AFV_M3	AFV_M4	AFV_M5	AFV_M6
RF MDI importance	**Caudate**	0.007	0.911	0.035	0.020	0.002	0.007	0.000	0.004	0.013	0.000	0.000
	**lentiform**	0.068	0.242	0.212	0.232	0.087	0.021	0.043	0.013	0.017	0.050	0.016
	**IC**	0.186	0.034	0.158	0.422	0.094	0.019	0.028	0.013	0.015	0.010	0.022
	**insula**	0.037	0.012	0.050	0.027	0.682	0.025	0.070	0.017	0.013	0.054	0.012
	**M1**	0.041	0.003	0.008	0.006	0.038	0.673	0.054	0.018	0.124	0.023	0.012
	**M2**	0.033	0.013	0.018	0.020	0.055	0.052	0.679	0.037	0.019	0.047	0.027
	**M3**	0.083	0.012	0.021	0.020	0.032	0.026	0.102	0.443	0.023	0.054	0.184
	**M4**	0.049	0.004	0.016	0.010	0.010	0.033	0.029	0.014	0.796	0.029	0.010
	**M5**	0.091	0.007	0.010	0.011	0.007	0.020	0.038	0.014	0.038	0.723	0.042
	**M6**	0.081	0.014	0.021	0.021	0.031	0.031	0.073	0.239	0.024	0.051	0.414
RF permutation importance in the training set	**ROI**	**stroke volume log ml**	**AFV_Caudate**	**AFV_lentiform**	**AFV_IC**	**AFV_insula**	**AFV_M1**	**AFV_M2**	**AFV_M3**	**AFV_M4**	**AFV_M5**	**AFV_M6**
	**Caudate**	0.000	0.250	0.073	0.000	0.000	0.000	0.000	0.000	0.000	0.000	0.000
	**lentiform**	0.029	0.017	0.181	0.050	0.029	0.002	0.013	0.002	0.003	0.013	0.000
	**IC**	0.091	0.013	0.060	0.211	0.080	-0.005	0.024	0.005	0.006	0.015	0.006
	**insula**	0.008	0.000	0.031	0.005	0.466	0.014	0.078	0.012	0.002	0.020	0.001
	**M1**	0.012	0.004	0.003	0.003	0.007	0.421	0.029	0.000	0.140	0.010	0.002
	**M2**	0.023	0.000	0.009	0.008	0.026	0.040	0.500	0.020	0.008	0.033	0.012
	**M3**	0.001	0.000	0.002	0.002	0.000	0.004	0.002	0.475	0.000	0.005	0.013
	**M4**	0.026	0.007	0.013	0.006	0.009	0.006	0.008	0.002	0.511	0.013	0.000
	**M5**	0.052	0.000	0.005	0.002	0.000	0.005	0.050	0.002	0.010	0.524	0.027
	**M6**	0.000	0.000	0.004	0.003	0.005	0.006	0.004	0.032	0.000	0.003	0.317
RF permutation importance in the testing set	**ROI**	**stroke volume log ml**	**AFV_Caudate**	**AFV_lentiform**	**AFV_IC**	**AFV_insula**	**AFV_M1**	**AFV_M2**	**AFV_M3**	**AFV_M4**	**AFV_M5**	**AFV_M6**
	**Caudate**	0.000	0.220	0.051	0.000	0.000	0.000	0.000	0.000	0.000	0.000	0.000
	**lentiform**	0.040	0.026	0.128	0.025	0.028	0.008	0.018	0.003	0.008	0.017	0.008
	**IC**	0.048	0.003	0.033	0.176	0.039	0.000	0.000	0.000	0.014	0.000	-0.006
	**insula**	0.004	0.005	0.023	0.013	0.453	0.000	0.122	0.008	0.000	0.039	0.000
	**M1**	0.007	0.000	0.009	0.000	0.003	0.451	-0.011	0.001	0.069	0.000	0.000
	**M2**	0.012	0.000	0.000	-0.001	-0.024	-0.017	0.408	-0.001	0.000	-0.003	-0.004
	**M3**	0.000	0.000	0.000	0.000	0.000	-0.002	0.000	0.462	0.000	0.000	0.000
	**M4**	-0.012	0.000	0.000	0.000	0.004	0.000	-0.016	0.006	0.466	0.012	0.000
	**M5**	0.015	0.000	0.000	0.000	0.000	-0.001	0.074	0.000	0.015	0.433	0.007
	**M6**	-0.002	-0.002	-0.001	-0.002	0.000	-0.010	0.000	0.033	-0.014	-0.009	0.333

Supplementary Table 5. Feature importance as revealed by the analysis of Mean Decrease in Impurity (MDI) and permutation test of the Random Forest (RF) model.

BT		
param_grid	Best model parameters	
{’th’:range(0,1,0.01)}	Caudate	{’th’: 0.107494}
	lentiform	{’th’: 0.070096}
	IC	{’th’: 0.35373200000000005}
	insula	{’th’: 0.12784399999999999}
	M1	{’th’: 0.029374000000000008}
	M2	{’th’: 0.14613800000000002}
	M3	{’th’: 0.10804200000000003}
	M4	{’th’: 0.04074200000000001}
	M5	{’th’: 0.097524}
	M6	{’th’: 0.107554}
RF		
param_grid	Best model parameters	
{’criterion’ : [’gini’],<br>’max_features’ : [’sqrt’, None],<br>’max_depth’: [None,2,4,6,8,10],<br>’n_estimators’ :[10,25,50,100,250,500,750,1000]}	Caudate	{’criterion’: ’gini’, ’max_depth’: 2, ’max_features’: None, ’n_estimators’: 100}
	lentiform	{’criterion’: ’gini’, ’max_depth’: 6, ’max_features’: ’sqrt’, ’n_estimators’: 250}
	IC	{’criterion’: ’gini’, ’max_depth’: None, ’max_features’: None, ’n_estimators’: 250}
	insula	{’criterion’: ’gini’, ’max_depth’: 2, ’max_features’: None, ’n_estimators’: 750}
	M1	{’criterion’: ’gini’, ’max_depth’: 8, ’max_features’: ’sqrt’, ’n_estimators’: 500}
	M2	{’criterion’: ’gini’, ’max_depth’: 10, ’max_features’: ’sqrt’, ’n_estimators’: 750}
	M3	{’criterion’: ’gini’, ’max_depth’: 6, ’max_features’: None, ’n_estimators’: 250}
	M4	{’criterion’: ’gini’, ’max_depth’: 10, ’max_features’: None, ’n_estimators’: 750}
	M5	{’criterion’: ’gini’, ’max_depth’: 8, ’max_features’: ’sqrt’, ’n_estimators’: 500}
	M6	{’criterion’: ’gini’, ’max_depth’: 4, ’max_features’: None, ’n_estimators’: 50}
KNN		
param_grid	Best model parameters	
{’n_neighbors’:[2,3,4,5,6,7,8,9,10,11,12,13,14,15,16,17,18,19,20,<br>25,30,35,40,45,50,60,70,80,90,100,150,200],<br>’weights’ : [’uniform’, ’distance’]}	Caudate	{’n_neighbors’: 6, ’weights’: ’distance’}
	lentiform	{’n_neighbors’: 6, ’weights’: ’distance’}
	IC	{’n_neighbors’: 6, ’weights’: ’distance’}
	insula	{’n_neighbors’: 10, ’weights’: ’distance’}
	M1	{’n_neighbors’: 4, ’weights’: ’distance’}
	M2	{’n_neighbors’: 15, ’weights’: ’distance’}
	M3	{’n_neighbors’: 5, ’weights’: ’uniform’}
	M4	{’n_neighbors’: 2, ’weights’: ’distance’}
	M5	{’n_neighbors’: 7, ’weights’: ’uniform’}
	M6	{’n_neighbors’: 3, ’weights’: ’uniform’}
SVM		
param_grid	Best model parameters	
{’kernel’: [’rbf’],<br>’gamma’: [1e-3, 1e-4, 1e-5],<br>’C’: [1,2,3,4,5,6,7,8,9,10,15,20,25,30, 50, 75, 100, 200, 300, 500]},<br>{’kernel’: [’linear’],<br>’C’: [1,2,3,4,5,6,7,8,9,10,15,20,25,30, 50, 75, 100, 200, 300, 500]}	Caudate	{’C’: 1, ’kernel’: ’linear’}
	lentiform	{’C’: 2, ’kernel’: ’linear’}
	IC	{’C’: 25, ’kernel’: ’linear’}
	insula	{’C’: 300, ’kernel’: ’linear’}
	M1	{’C’: 50, ’kernel’: ’linear’}
	M2	{’C’: 100, ’kernel’: ’linear’}
	M3	{’C’: 75, ’kernel’: ’linear’}
	M4	{’C’: 20, ’kernel’: ’linear’}
	M5	{’C’: 200, ’kernel’: ’linear’}
	M6	{’C’: 100, ’kernel’: ’linear’}
MLP		
param_grid	Best model parameters	
{’hidden_layer_sizes’ : [64,128,256,512,1024],<br><br>’alpha’: [1E-5, 1E-4, 1E-3],<br>’max_iter’ : [200,500,800],<br>’learning_rate_init’ : [1E-4,1E-3]}	Caudate	{’alpha’: 0.0001, ’hidden_layer_sizes’: 512, ’learning_rate_init’: 0.001, ’max_iter’: 500}
	lentiform	{’alpha’: 1e-05, ’hidden_layer_sizes’: 1024, ’learning_rate_init’: 0.001, ’max_iter’: 500}
	IC	{’alpha’: 0.001, ’hidden_layer_sizes’: 1024, ’learning_rate_init’: 0.001, ’max_iter’: 800}
	insula	{’alpha’: 0.0001, ’hidden_layer_sizes’: 1024, ’learning_rate_init’: 0.001, ’max_iter’: 200}
	M1	{’alpha’: 0.001, ’hidden_layer_sizes’: 256, ’learning_rate_init’: 0.001, ’max_iter’: 500}
	M2	{’alpha’: 0.0001, ’hidden_layer_sizes’: 1024, ’learning_rate_init’: 0.001, ’max_iter’: 500}
	M3	{’alpha’: 0.001, ’hidden_layer_sizes’: 512, ’learning_rate_init’: 0.001, ’max_iter’: 500}
	M4	{’alpha’: 1e-05, ’hidden_layer_sizes’: 512, ’learning_rate_init’: 0.001, ’max_iter’: 500}
	M5	{’alpha’: 0.001, ’hidden_layer_sizes’: 128, ’learning_rate_init’: 0.001, ’max_iter’: 800}
	M6	{’alpha’: 0.0001, ’hidden_layer_sizes’: 1024, ’learning_rate_init’: 0.001, ’max_iter’: 500}

Supplementary Table 6. Table of hyperparameters for the ML models for ASPECTS prediction via using lesion segmentation from ADS pipeline.

ROI	Number of occurrences	Metric	LDA	QDA	KNN	SVM	RF	MLP	BT	Ensemble all majority	Ensemble top5 majority	Ensemble top3 majority
Caudate	72	BACC	0.829	0.878	0.851	0.852	0.907	0.870	0.921	0.913	0.913	0.913
		F1	0.771	0.791	0.789	0.801	0.851	0.812	0.849	0.873	0.873	0.873
		Precision	0.905	0.746	0.852	0.906	0.844	0.857	0.799	0.886	0.886	0.886
		Sensitivity	0.683	0.853	0.745	0.729	0.869	0.784	0.921	0.861	0.861	0.861
lentiform	128	BACC	0.801	0.803	0.857	0.854	0.879	0.878	0.763	0.928	0.922	0.938
		F1	0.764	0.771	0.836	0.831	0.861	0.860	0.726	0.916	0.909	0.927
		Precision	0.829	0.793	0.835	0.831	0.833	0.842	0.740	0.896	0.882	0.910
		Sensitivity	0.717	0.758	0.841	0.838	0.895	0.883	0.722	0.938	0.938	0.945
IC	49	BACC	0.630	0.678	0.655	0.607	0.637	0.650	0.664	0.819	0.819	0.833
		F1	0.387	0.451	0.442	0.338	0.398	0.426	0.430	0.753	0.753	0.786
		Precision	0.553	0.437	0.669	0.590	0.536	0.599	0.443	0.889	0.889	0.943
		Sensitivity	0.313	0.489	0.346	0.250	0.334	0.352	0.472	0.653	0.653	0.673
insula	174	BACC	0.857	0.824	0.824	0.883	0.877	0.874	0.847	0.926	0.914	0.919
		F1	0.874	0.841	0.857	0.893	0.900	0.889	0.862	0.932	0.920	0.926
		Precision	0.895	0.875	0.846	0.926	0.891	0.908	0.895	0.958	0.951	0.952
		Sensitivity	0.857	0.813	0.870	0.865	0.912	0.874	0.835	0.908	0.891	0.902
M1	83	BACC	0.822	0.858	0.800	0.871	0.890	0.869	0.904	0.909	0.905	0.897
		F1	0.759	0.782	0.723	0.822	0.833	0.819	0.832	0.877	0.866	0.862
		Precision	0.869	0.752	0.818	0.865	0.819	0.863	0.764	0.899	0.877	0.896
		Sensitivity	0.685	0.823	0.659	0.791	0.856	0.789	0.921	0.855	0.855	0.831
M2	107	BACC	0.801	0.818	0.782	0.822	0.829	0.828	0.807	0.911	0.909	0.922
		F1	0.746	0.763	0.718	0.773	0.780	0.779	0.749	0.890	0.886	0.911
		Precision	0.855	0.735	0.823	0.831	0.807	0.813	0.725	0.912	0.903	0.968
		Sensitivity	0.668	0.801	0.644	0.729	0.764	0.755	0.787	0.869	0.869	0.860
M3	93	BACC	0.856	0.871	0.841	0.894	0.914	0.886	0.910	0.934	0.942	0.937
		F1	0.821	0.817	0.790	0.861	0.875	0.850	0.860	0.917	0.924	0.922
		Precision	0.947	0.800	0.860	0.899	0.860	0.888	0.811	0.943	0.934	0.954
		Sensitivity	0.731	0.842	0.738	0.832	0.896	0.822	0.921	0.892	0.914	0.892
M4	90	BACC	0.805	0.844	0.785	0.846	0.854	0.844	0.854	0.921	0.910	0.916
		F1	0.743	0.773	0.698	0.798	0.796	0.791	0.777	0.902	0.876	0.895
		Precision	0.894	0.742	0.706	0.878	0.815	0.855	0.729	0.940	0.886	0.939
		Sensitivity	0.646	0.816	0.699	0.740	0.788	0.745	0.856	0.867	0.867	0.856
M5	137	BACC	0.836	0.785	0.811	0.870	0.883	0.870	0.854	0.900	0.908	0.928
		F1	0.811	0.766	0.797	0.854	0.873	0.856	0.845	0.891	0.900	0.922
		Precision	0.904	0.766	0.781	0.905	0.877	0.881	0.802	0.922	0.917	0.939
		Sensitivity	0.740	0.773	0.819	0.813	0.873	0.838	0.898	0.861	0.883	0.905
M6	93	BACC	0.859	0.856	0.889	0.873	0.889	0.880	0.880	0.932	0.922	0.937
		F1	0.822	0.798	0.854	0.832	0.847	0.836	0.818	0.912	0.892	0.918
		Precision	0.929	0.790	0.892	0.874	0.856	0.854	0.766	0.933	0.892	0.933
		Sensitivity	0.744	0.813	0.824	0.802	0.845	0.826	0.885	0.892	0.892	0.903
Average of ROIs		BACC	0.810	0.822	0.809	0.837	0.856	0.845	0.840	0.909	0.906	0.914
		F1	0.750	0.755	0.750	0.780	0.801	0.792	0.775	0.886	0.880	0.894
		Precision	0.858	0.744	0.808	0.851	0.814	0.836	0.747	0.918	0.902	0.932
		Sensitivity	0.678	0.778	0.719	0.739	0.803	0.767	0.822	0.860	0.862	0.863

Supplementary Table 7. Performance of the ML models according to cross validation on the training set via using lesion segmentation from ADS pipeline.

ROI	Number of occurrences	Metric	LDA	QDA	KNN	SVM	RF	MLP	BT	Ensemble	Inter-annotators
Caudate	36	BACC	0.810	0.846	0.820	0.816	0.836	0.816	0.854	0.830	0.901
		F1	0.762	0.800	0.771	0.769	0.794	0.769	0.811	0.788	0.882
		Precision	0.889	0.769	0.794	0.862	0.844	0.862	0.789	0.867	0.938
		Sensitivity	0.667	0.833	0.750	0.694	0.750	0.694	0.833	0.722	0.833
		Kappa	0.656	0.680	0.649	0.660	0.689	0.660	0.700	0.685	0.822
lentiform	59	BACC	0.868	0.843	0.855	0.869	0.885	0.881	0.808	0.869	0.903
		F1	0.889	0.874	0.881	0.902	0.919	0.909	0.818	0.902	0.899
		Precision	0.897	0.867	0.881	0.873	0.877	0.887	0.882	0.873	0.980
		Sensitivity	0.881	0.881	0.881	0.932	0.966	0.932	0.763	0.932	0.831
		Kappa	0.732	0.689	0.711	0.748	0.789	0.770	0.599	0.748	0.780
IC	23	BACC	0.641	0.735	0.757	0.639	0.648	0.750	0.685	0.676	0.768
		F1	0.444	0.577	0.650	0.438	0.457	0.634	0.510	0.514	0.638
		Precision	0.615	0.517	0.765	0.778	0.667	0.722	0.464	0.750	0.625
		Sensitivity	0.348	0.652	0.565	0.304	0.348	0.565	0.565	0.391	0.652
		Kappa	0.334	0.431	0.565	0.354	0.355	0.542	0.344	0.423	0.527
insula	54	BACC	0.935	0.864	0.919	0.952	0.918	0.952	0.899	0.952	0.944
		F1	0.931	0.863	0.926	0.952	0.927	0.952	0.907	0.952	0.941
		Precision	1.000	0.917	0.926	0.980	0.911	0.980	0.907	0.980	1.000
		Sensitivity	0.870	0.815	0.926	0.926	0.944	0.926	0.907	0.926	0.889
		Kappa	0.861	0.721	0.839	0.900	0.838	0.900	0.799	0.900	0.880
M1	28	BACC	0.946	0.930	0.904	0.982	0.982	0.964	0.913	0.982	0.957
		F1	0.943	0.881	0.885	0.982	0.982	0.963	0.831	0.982	0.945
		Precision	1.000	0.839	0.958	1.000	1.000	1.000	0.730	1.000	0.963
		Sensitivity	0.893	0.929	0.821	0.964	0.964	0.929	0.964	0.964	0.929
		Kappa	0.923	0.832	0.844	0.975	0.975	0.949	0.752	0.975	0.925
M2	40	BACC	0.850	0.838	0.879	0.892	0.888	0.913	0.888	0.904	0.942
		F1	0.824	0.805	0.861	0.877	0.868	0.904	0.864	0.892	0.927
		Precision	1.000	0.745	0.969	0.970	0.917	1.000	0.854	0.971	0.905
		Sensitivity	0.700	0.875	0.775	0.800	0.825	0.825	0.875	0.825	0.950
		Kappa	0.737	0.656	0.784	0.807	0.788	0.850	0.772	0.829	0.876
M3	33	BACC	0.932	0.910	0.932	0.917	0.947	0.917	0.970	0.932	0.917
		F1	0.921	0.870	0.921	0.903	0.925	0.903	0.943	0.921	0.903
		Precision	0.967	0.833	0.967	0.966	0.912	0.966	0.892	0.967	0.966
		Sensitivity	0.879	0.909	0.879	0.848	0.939	0.848	1.000	0.879	0.848
		Kappa	0.884	0.801	0.884	0.860	0.888	0.860	0.912	0.884	0.860
M4	29	BACC	0.848	0.819	0.813	0.865	0.858	0.875	0.896	0.865	0.828
		F1	0.808	0.730	0.737	0.830	0.815	0.836	0.847	0.830	0.792
		Precision	0.913	0.676	0.750	0.917	0.880	0.885	0.833	0.917	1.000
		Sensitivity	0.724	0.793	0.724	0.759	0.759	0.793	0.862	0.759	0.655
		Kappa	0.741	0.607	0.632	0.770	0.747	0.775	0.784	0.770	0.730
M5	41	BACC	0.840	0.820	0.846	0.865	0.881	0.856	0.859	0.869	0.888
		F1	0.811	0.791	0.818	0.842	0.861	0.831	0.833	0.846	0.867
		Precision	0.909	0.720	0.766	0.914	0.895	0.889	0.814	0.892	0.857
		Sensitivity	0.732	0.878	0.878	0.780	0.829	0.780	0.854	0.805	0.878
		Kappa	0.702	0.620	0.677	0.746	0.770	0.726	0.713	0.748	0.773
M6	32	BACC	0.883	0.916	0.892	0.907	0.924	0.892	0.924	0.923	0.953
		F1	0.862	0.879	0.867	0.885	0.892	0.867	0.892	0.903	0.951
		Precision	0.962	0.853	0.929	0.931	0.879	0.929	0.879	0.933	1.000
		Sensitivity	0.781	0.906	0.813	0.844	0.906	0.813	0.906	0.875	0.906
		Kappa	0.807	0.819	0.810	0.835	0.840	0.810	0.840	0.860	0.929
Total ASPECTS	100	BACC	0.343	0.285	0.407	0.450	0.368	0.368	0.385	0.408	0.480
		F1	0.466	0.339	0.458	0.571	0.454	0.498	0.350	0.538	0.594
		Precision	0.483	0.394	0.486	0.582	0.473	0.536	0.453	0.542	0.659
		Sensitivity	0.470	0.320	0.460	0.580	0.450	0.500	0.330	0.550	0.580
		Kappa	0.375	0.232	0.365	0.502	0.360	0.413	0.261	0.467	0.510
		Kappa weighted	0.715	0.635	0.720	0.784	0.768	0.744	0.715	0.768	0.796
Total ASPECTS with tolerance	100	BACC	0.705	0.656	0.769	0.822	0.842	0.777	0.808	0.797	0.849
		F1	0.808	0.725	0.826	0.882	0.887	0.845	0.815	0.854	0.887
		Precision	0.822	0.770	0.837	0.890	0.899	0.859	0.862	0.862	0.919
		Sensitivity	0.810	0.720	0.830	0.880	0.890	0.850	0.800	0.860	0.880
		Kappa	0.773	0.673	0.798	0.857	0.870	0.822	0.766	0.834	0.859
		Kappa weighted	0.827	0.748	0.849	0.889	0.914	0.868	0.842	0.875	0.899

Supplementary Table 8. Comparison of performance of ML models and inter-annotators in the external testing set (n=100) via using lesion segmentation from ADS pipeline. For the total aspects "whit tolerance", predicted ASPECTS within ± 1 difference from the ground true ASPECTS were considered true positives.

SUPPLEMENTARY FIGURES

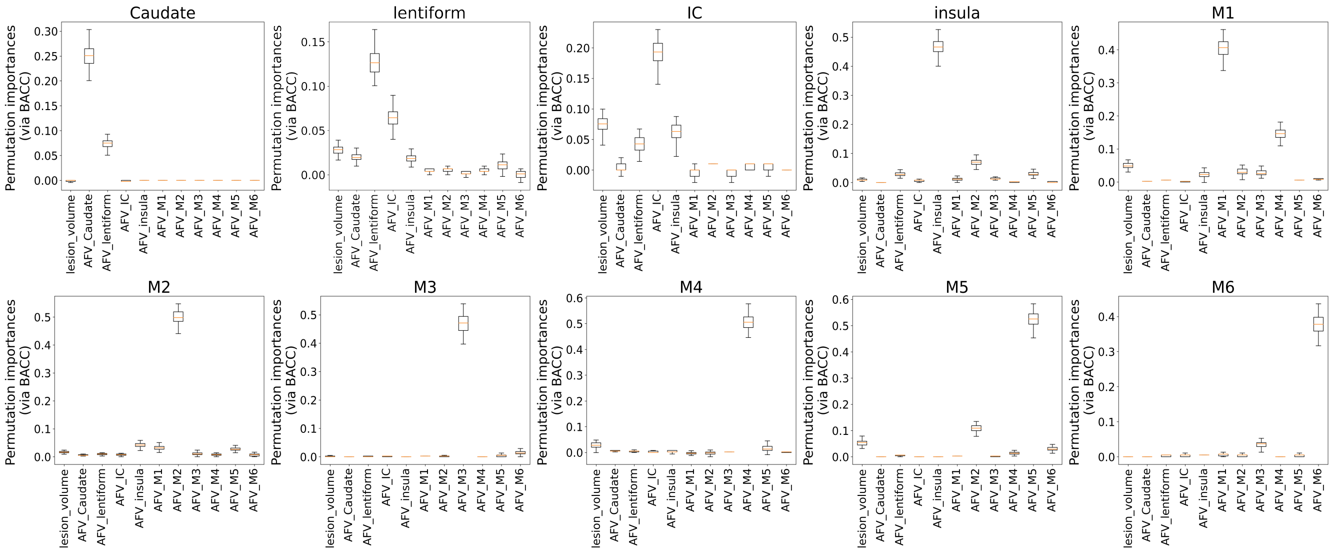

Supplementary Figure 1. Feature importance analysis of the random forest (RF) models accessed by permutation test, in the training set.

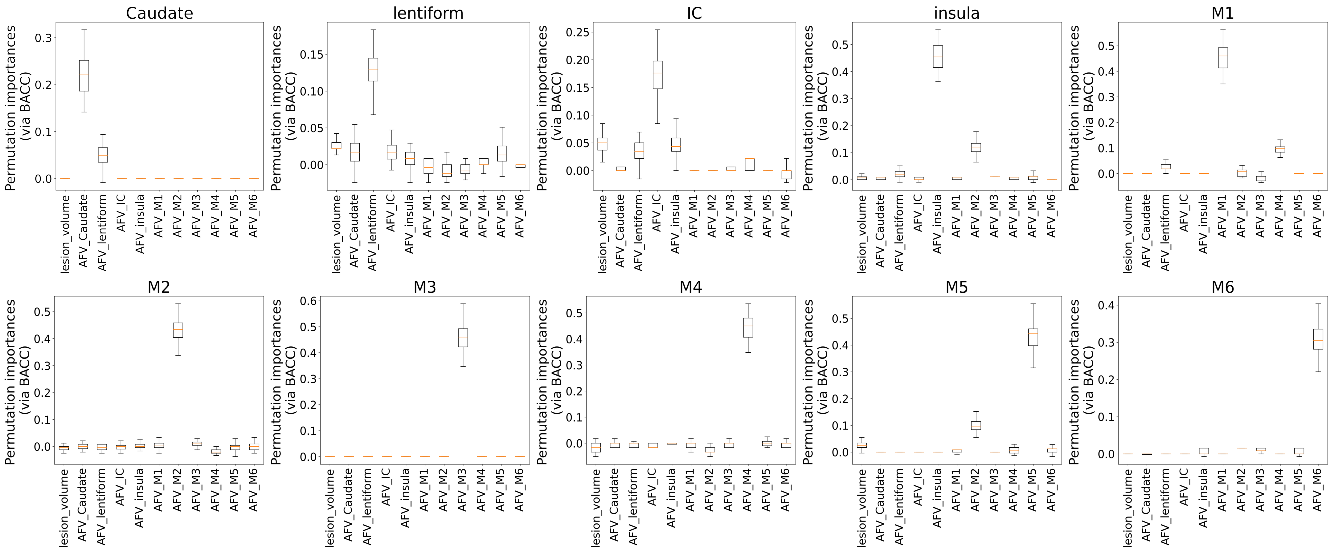

Supplementary Figure 2. Feature importance analysis of the random forest (RF) models accessed by permutation test, in the testing set

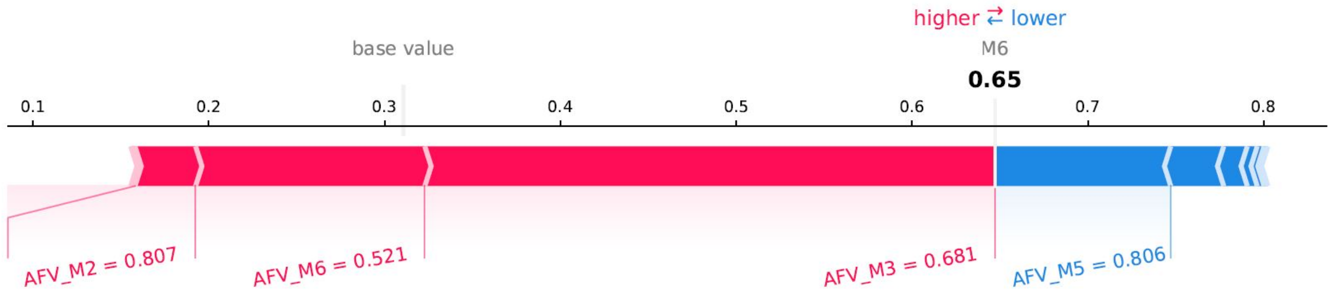

Supplementary Figure 3. Illustrative example of the output of our interpretable pre-trained models in ADS. This example shows how the model interprets the contribution of each AFV component to predict the M6 score.
